# Auxiliary Diagnosis of Children With Attention-Deficit/Hyperactivity Disorder Using Eye-Tracking and Digital Biomarkers: Case-Control Study

**DOI:** 10.2196/58927

**Published:** 2024-11-29

**Authors:** Zhongling Liu, Jinkai Li, Yuanyuan Zhang, Dan Wu, Yanyan Huo, Jianxin Yang, Musen Zhang, Chuanfei Dong, Luhui Jiang, Ruohan Sun, Ruoyin Zhou, Fei Li, Xiaodan Yu, Daqian Zhu, Yao Guo, Jinjin Chen

**Affiliations:** 1 Child Health Care Medical Division Shanghai Children's Hospital School of Medicine, Shanghai Jiao Tong University Shanghai China; 2 Institute of Medical Robotics School of Biomedical Engineering Shanghai Jiao Tong University Shanghai China; 3 School of Medicine Shanghai Jiao Tong University Shanghai China; 4 Department of Developmental and Behavioural Pediatric & Child Primary Care Xinhua Hospital School of Medicine, Shanghai Jiao Tong University Shanghai China; 5 Department of Developmental and Behavioral Pediatrics Shanghai Children's Medical Center School of Medicine, Shanghai Jiao Tong University Shanghai China; 6 Department of Psychiatry Children's Hospital of Fudan University Shanghai China

**Keywords:** attention deficit disorder with hyperactivity, eye-tracking, auxiliary diagnosis, digital biomarker, antisaccade, machine learning

## Abstract

**Background:**

Attention-deficit/hyperactivity disorder (ADHD) is a common neurodevelopmental disorder in school-aged children. The lack of objective biomarkers for ADHD often results in missed diagnoses or misdiagnoses, which lead to inappropriate or delayed interventions. Eye-tracking technology provides an objective method to assess children’s neuropsychological behavior.

**Objective:**

The aim of this study was to develop an objective and reliable auxiliary diagnostic system for ADHD using eye-tracking technology. This system would be valuable for screening for ADHD in schools and communities and may help identify objective biomarkers for the clinical diagnosis of ADHD.

**Methods:**

We conducted a case-control study of children with ADHD and typically developing (TD) children. We designed an eye-tracking assessment paradigm based on the core cognitive deficits of ADHD and extracted various digital biomarkers that represented participant behaviors. These biomarkers and developmental patterns were compared between the ADHD and TD groups. Machine learning (ML) was implemented to validate the ability of the extracted eye-tracking biomarkers to predict ADHD. The performance of the ML models was evaluated using 5-fold cross-validation.

**Results:**

We recruited 216 participants, of whom 94 (43.5%) were children with ADHD and 122 (56.5%) were TD children. The ADHD group showed significantly poorer performance (for accuracy and completion time) than the TD group in the prosaccade, antisaccade, and delayed saccade tasks. In addition, there were substantial group differences in digital biomarkers, such as pupil diameter fluctuation, regularity of gaze trajectory, and fixations on unrelated areas. Although the accuracy and task completion speed of the ADHD group increased over time, their eye-movement patterns remained irregular. The TD group with children aged 5 to 6 years outperformed the ADHD group with children aged 9 to 10 years, and this difference remained relatively stable over time, which indicated that the ADHD group followed a unique developmental pattern. The ML model was effective in discriminating the groups, achieving an area under the curve of 0.965 and an accuracy of 0.908.

**Conclusions:**

The eye-tracking biomarkers proposed in this study effectively identified differences in various aspects of eye-movement patterns between the ADHD and TD groups. In addition, the ML model constructed using these digital biomarkers achieved high accuracy and reliability in identifying ADHD. Our system can facilitate early screening for ADHD in schools and communities and provide clinicians with objective biomarkers as a reference.

## Introduction

### Background

Attention-deficit/hyperactivity disorder (ADHD) is a common neurodevelopmental disorder in school-aged children, characterized by deficits in attention, hyperactivity, and impulsivity. Globally, the estimated prevalence of ADHD in children and adolescents is approximately 5.29% [1]; in China, the prevalence is approximately 6.4% [2]. People with ADHD typically exhibit deficiencies in various cognitive domains, and these symptoms can persist into adolescence and adulthood, which can result in academic underachievement and societal issues, such as substance abuse and violence [3]. Therefore, early identification, diagnosis, and intervention for ADHD are essential.

Despite recent advances, the diagnosis of ADHD relies heavily on subjective judgments based on the observations of children’s behavior. Consequently, this can lead to both over and underdiagnosis, as well as inappropriate treatments. Therefore, there is an urgent need to develop methods to identify reliable ADHD biomarkers. Furthermore, given that poor academic performance is the most common concern of individuals with ADHD, it is crucial that we improve awareness and understanding of ADHD among parents and teachers to ensure timely identification of ADHD. However, on the one hand, most nonmedical professionals cannot be expected to gain specialized medical expertise, and on the other hand, physicians cannot frequently visit campuses to aid in ADHD assessment. This situation has resulted in delays in diagnosing children with ADHD. Developing mobile screening equipment will enable on-campus ADHD screening to facilitate timely identification and diagnosis of ADHD.

Eye-tracking technology is particularly suited for the assessment and diagnosis of ADHD because it offers an objective measurement of children’s neuropsychological behavior. Studies have shown that there is a significant overlap between the neural networks responsible for attention and those responsible for eye-movement control [4]. Children with ADHD experience difficulties with spatial perception and visual-motor integration [5], and these neurophysiological features associated with ADHD can be identified using eye-tracking assessments. In addition, children with ADHD often find lengthy and complex assessments challenging, particularly if they are required to wear additional equipment. Eye-tracking technology surpasses other neurophysiological techniques in its ability to record the neuropsychological activity of participants in a more natural setting [6]. This leads to better cooperation of children during assessments and higher reliability and generalizability of results.

Recent advances in computational psychiatry have enabled the extraction of eye-tracking metrics to discern behavioral alterations in children with ADHD [7-9]. These metrics encompass various aspects of visual attention, such as fixation duration, saccade velocity, and gaze entropy [10-12], which may serve as digital biomarkers for neurodevelopmental disorders [13,14]. By analyzing the temporal and spatial characteristics of eye movements, computational models can capture differences in visual behaviors between ADHD and typically developing (TD) children. Machine learning (ML) techniques have emerged as powerful tools for processing and interpreting large amounts of eye-tracking data [15-17]. Training ML models on labeled eye-tracking metrics has allowed the construction of robust and accurate classifiers to identify whether individuals belong to an ADHD or a TD group. Precise eye-tracking measurements and digital biomarkers hold great promise as objective and automated screening tools for ADHD, which will facilitate the development of early intervention strategies and improve the clinical outcomes of affected children [7,18,19]. Moreover, the evolution of mobile eye-tracking technology and devices, coupled with portable computing sources, such as smartphones and tablets, will allow the implementation of eye-tracking assessments in various scenarios and thus address the need for ADHD screening in the community [20-22].

### Related Work

Neuroimaging studies have shown that children with ADHD have multidimensional brain function abnormalities. The impairment of inhibitory control is a fundamental factor contributing to cognitive and executive functioning deficiencies in individuals with ADHD [23]. However, these individuals also have motor coordination difficulties, poorer spatial perception [24-26], reduced auditory sensitivity, and problems with attentional integration of audiovisual stimuli [27].

Recently, there has been a growing interest in exploring the use of eye-tracking technology to study the neurophysiological features of ADHD. A meta-analysis of the various behavioral tests developed over the last 5 decades to evaluate eye movement and cognitive control [28] revealed that eye-tracking evaluations of children with ADHD yielded the most reliable and consistent outcomes when eliminating bias. Most of these tests focused on saccade, which is one of the most crucial type of eye movement. Children with ADHD perform significantly worse than TD children across all tasks, with greater variability for each metric in the antisaccade task [29].

To ensure that the screening method is appropriate for children with ADHD, we must use a paradigm that is brief and simple to perform yet capable of highlighting cognitive deficits. In addition, the extracted eye-movement metrics should be able to comprehensively characterize children’s task performance. Several recent studies have used eye tracking to explore the characteristics of ADHD. Lemel et al [30] incorporated spoken-word recognition accuracy, gaze duration, and the number of transitions in response to a phonological competitor to analyze spoken-word processing in adverse listening conditions in individuals with ADHD. However, this paradigm was complex and required word recognition and was thus more suited to adult patients. Another study used a paradigm to assess children’s working memory; however, the task took 30 minutes to complete [31], which is not conducive to task completion in children with ADHD. Siqueiros et al [32] used the antisaccade task, which is a simple and reliable paradigm that suits children. However, only directional errors and expected eye movements were assessed; moreover, the paradigm was not sufficiently comprehensive to assess children’s task performance.

### Objectives

Studies conducted to date have provided valuable insight into automatic screening approaches for ADHD in children using eye-tracking devices. However, these studies have drawbacks that have hindered the development of a more robust and accurate auxiliary diagnostic system. For example, the paradigms were too time-consuming or complex for clinical ADHD screening, and the extracted metrics were not sufficiently comprehensive. ML models used in previous studies have typically achieved only modest accuracy and sensitivity, which limits clinical applicability. Furthermore, small sample sizes have limited the robustness of the results.

To address the aforementioned challenges, we aimed to develop an accurate and reliable auxiliary diagnostic system for ADHD in children using eye-tracking technology. Specifically, the objectives of this study were as follows:

1. To design an eye-tracking assessment paradigm that is easy to implement and can identify differences in eye-movement patterns between children with ADHD and TD children.
2. To extract effective eye-tracking metrics as digital biomarkers that quantitatively represent various aspects of eye-movement behaviors and use these biomarkers to construct and validate ML models to enable automatic screening of children for ADHD.
3. To achieve high accuracy and reliability of the ML model using a large dataset, which will facilitate early screening for ADHD and timely intervention for children with ADHD and thus contribute to improving the effectiveness of the health care system.

## Methods

### Participants

To ensure the representativeness of the ADHD and TD groups in this case-control study, we recruited participants from hospitals and schools separately. Children with ADHD were recruited from an outpatient clinic at a public pediatric hospital in Shanghai, China, whereas TD children were recruited from 2 general public elementary schools in Shanghai (one from an urban area and another from a suburban area). The children were divided into 3 age groups: group 1 (5-6 years), group 2 (7-8 years), and group 3 (9-10 years).

The inclusion criteria for the ADHD group were children in grades 1 to 3 with a clinical diagnosis of ADHD who were not currently receiving treatment. The inclusion criteria for the TD group were children in grades 1 to 3 with a negative assessment on the Swanson, Nolan, and Pelham Rating Scale (SNAP-IV) [33].

The exclusion criteria were children with a full-scale score of <75 on the Wechsler Intelligence Scale for Children; children who had a history of severe traumatic brain injury, neurological disorders, severe physical illnesses, and psychiatric illnesses (eg, mood disorders and schizophrenia); and those unable to undergo eye-tracking examinations.

From December 2022 to April 2023, a total of 100 children with a clinical diagnosis of ADHD were recruited. Of these, 4 participants with a history of severe traumatic brain injury, neurological disorders, and other severe physical and psychiatric disorders and 2 participants who were unable to tolerate the eye-tracking assessment were excluded. This resulted in 94 participants in the ADHD group.

A total of 150 children were randomly selected as the TD group. Of these, 15 children refused to participate in the program. In addition, 2 children with a history of severe traumatic brain injury, neurological disorders, and other severe physical and psychiatric disorders and 11 children who were considered to have ADHD after the interviews and evaluations were excluded. Finally, 122 children were included in the study as the TD control group.

All personnel involved in administering the assessments in this study were full-time child health practitioners who had been working in child health care for more than 3 years. Standardized survey administration training was provided before the tests were administered.

### Ethical Considerations

Before the assessment began, the purpose of the project was explained to the children and their guardians, and written informed consent was obtained from the guardians. All participants could withdraw at any stage of the study. Interviews were then conducted with the guardians to gather data on the basic conditions of the children. Children who fulfilled the inclusion and exclusion criteria were formally enrolled in the study and underwent the SNAP-IV and eye-tracking assessments. All data will be stored in a deidentified form. No participants will receive any benefit from participating in this study, but they will receive a booklet reporting the results of the assessments involved in this study as a souvenir.

The study protocol and informed consent form were approved by the Shanghai Children’s Hospital Institutional Review Board (2022R126-F01).

### Paradigm Design

#### Overview

Eye movements were recorded at a sampling rate of 1200 Hz using the Tobii Pro Spectrum eye tracker (Tobii Pro AB), a screen-based eye tracker that captures eye movements and pupillary responses. Visual stimuli were presented at a screen response rate of <5 milliseconds on a 24-inch monitor with a resolution of 1920×1080 pixels (16:9 ratio). The Tobii Pro Lab software (version 1.194; Tobii Pro AB) was used to set up the experiment.

**Figure 1:**
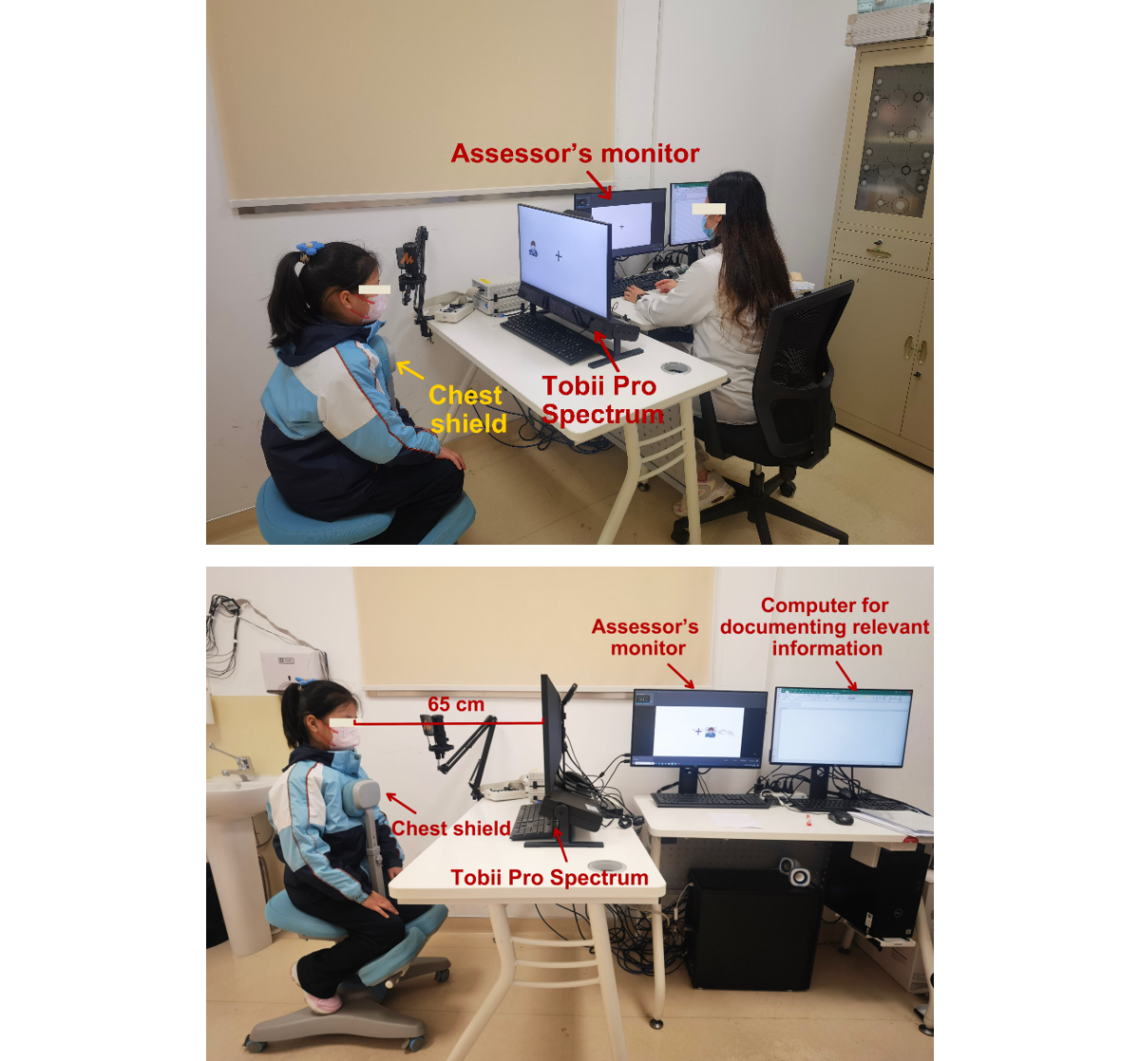
Eye-tracking assessment scenario settings.

The assessment procedure was performed in a quiet room with only 1 overhead light source (Figure 1). Participants were seated in a special seat with a chest shield to limit upper body movement and help stabilize the head. The cushion was adjusted to ensure that the center of the screen was at the same level as the participant’s head. The participant was seated in a position in which they were unable to observe the assessor’s screen or operations to minimize distractions. Participants maintained a distance of 65 cm from the screen and began the formal assessment following a 5-point calibration. Before each task, a prompt screen appeared, and the assessor provided detailed instructions to ensure that the participant fully understood the task content before proceeding with formal testing.

**Figure 2:**
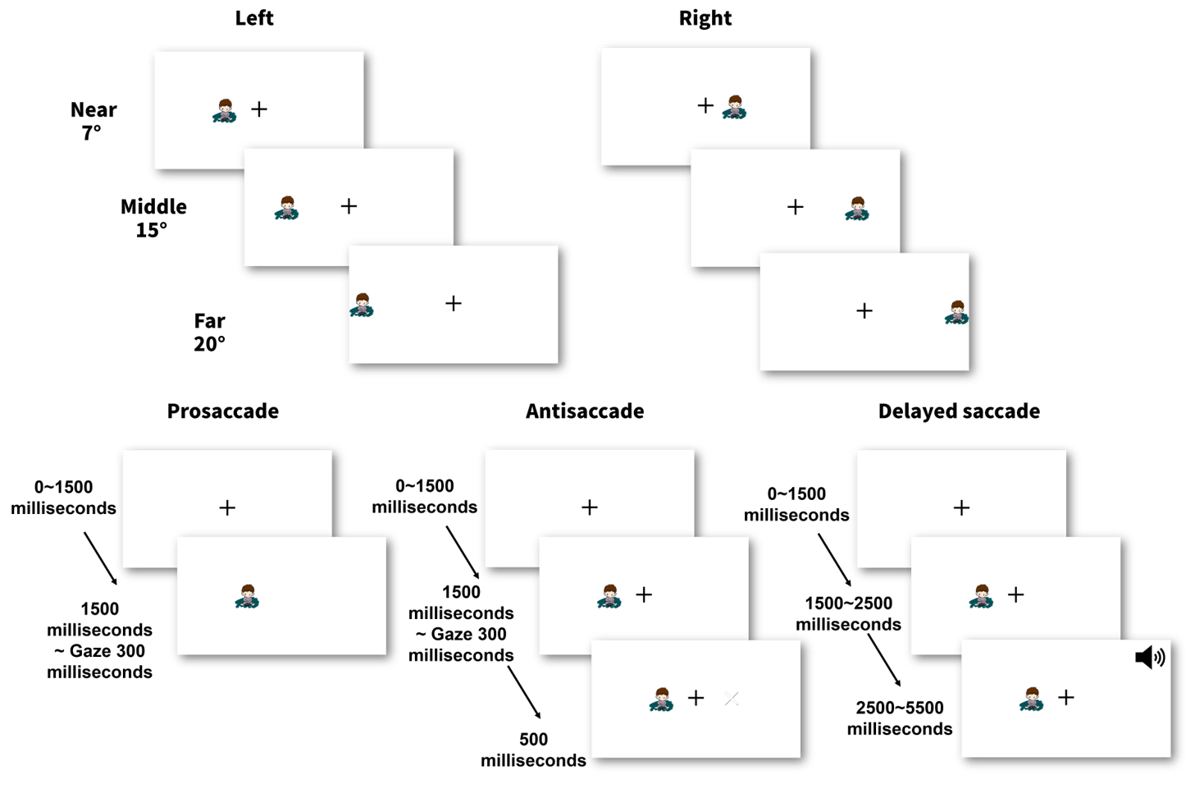
The eye-tracking assessment paradigm.

During the assessment, participants were asked to complete 3 saccade tasks sequentially (Figure 2): prosaccade, antisaccade, and delayed saccade. The stimulus was 5 cm high and 5 cm wide and randomly appeared on the left or right side of the screen. There was a central fixation cross in the middle of the screen, and the stimuli were set at 7°, 15°, and 20° away from the central cross for the different eccentricities. For each trial, a stimulus would randomly appear twice at one of the aforementioned 6 positions.

#### Prosaccade Task

Prosaccade, also known as reflexive saccade or visually guided saccade, is an abrupt eye movement triggered by the sudden appearance of a stimulus [34]. It is primarily induced by exogenous stimuli and serves as a baseline measure. In the prosaccade task, participants were instructed to initially fixate on the central fixation cross. After 1500 milliseconds, a stimulus appeared randomly in one of the aforementioned 6 positions. Participants were required to quickly shift their gaze toward the stimulus. Once participants fixated on the stimulus area (SA) for more than 300 milliseconds, the next trial was started automatically.

#### Antisaccade Task

In the antisaccade task, participants were required to first fixate on the central fixation cross. After 1500 milliseconds, 1 stimulus appeared randomly in one of the 6 aforementioned positions. Participants were required to quickly shift their gaze to the target area (TA), which was the location symmetrically opposite to the stimulus relative to the central fixation cross. Upon maintaining fixation at the TA for more than 300 milliseconds, a white feedback cross automatically appeared at the TA position to indicate success before proceeding to the next trial. If the participant decided to abandon the trial, the assessor pressed the space bar to skip the trial, and a white cross was displayed at the TA before moving on to the next trial. Previous studies have used a paradigm in which the central fixation cross disappears when the stimulus is presented [28]. However, this can make accurately localizing the TA more challenging, which may result in children being unable to complete the task. Therefore, in this study, the central cross was retained to assist participants in locating the TA.

#### Delayed Saccade Task

The delayed saccade task, based on the go–no-go paradigm [35], was adapted to the cognitive abilities of children with ADHD. This task not only directly assesses inhibition but also requires participants to combine auditory discrimination and visuomotor modulation. Thus, the task assesses the multisensory integration and coordination capacity of individuals with ADHD. During the task, participants were instructed to fixate on the central fixation cross. After 1500 milliseconds, 1 stimulus appeared randomly in one of the 6 aforementioned positions. Participants were asked to maintain fixation on the central cross until they heard a sound cue after 1000 milliseconds, after which they were required to shift their gaze toward the SA as fast as possible. Then, after another 3000 milliseconds, the next trial was started automatically.

For each saccade task, there were 12 formal trials (2 trials for each position). Before the formal test, practice trials were provided, where stimuli were presented randomly in the 6 positions, to allow participants to familiarize themselves with the task.

### Area of Interest Division Across Tasks

**Figure 3:**
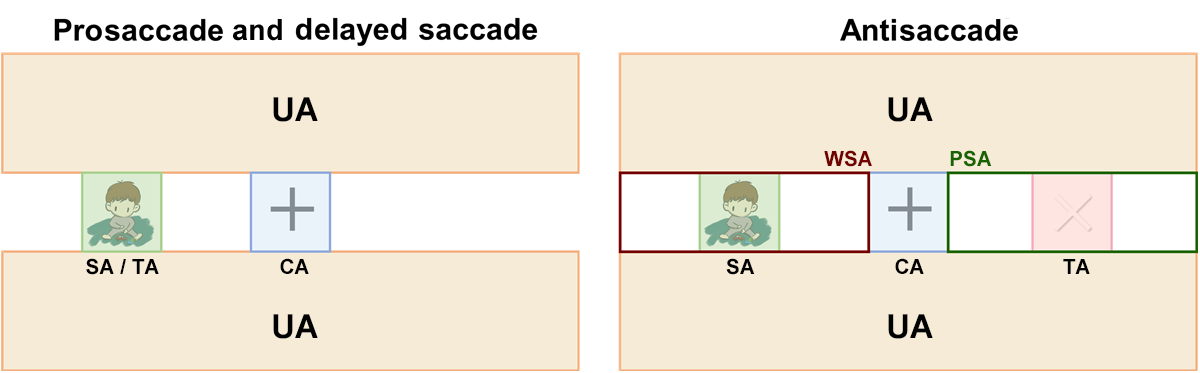
Illustration of the division of areas for extracting area-based eye-tracking metrics. CA: center area; PSA: proper-side area; SA: stimulus area; TA: target area; UA: unrelated area; WSA: wrong-side area.

**Figure 4:**
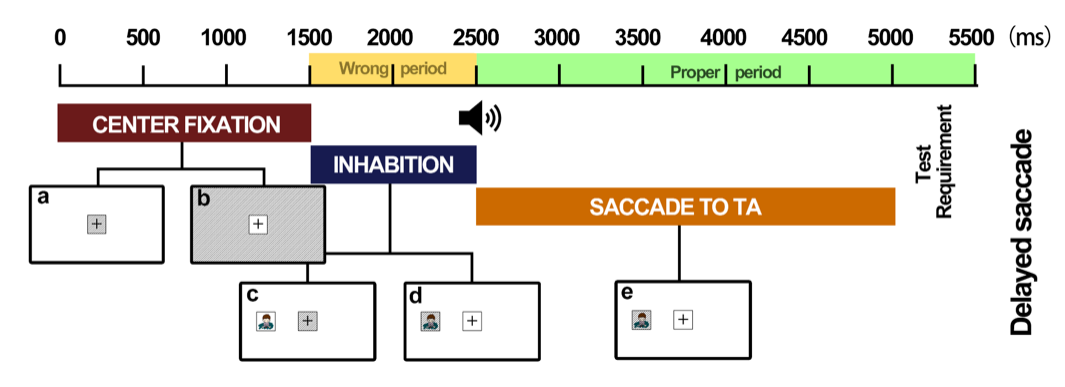
The different completion statuses in the delayed saccade task. From 0 to 1500 milliseconds, participants were asked to gaze at the center area (shaded area in a). If fixation fell into the shaded area in b, this indicated the occurrence of an intrusive saccade. From 1500 to 2500 milliseconds, participants were asked to maintain their fixation on the center area (shaded area in c) until they heard the cue. Thus, if fixation fell into the shaded area in d during this period, this was defined as a target area during the wrong period fixation (ie, saccade to the target area (TA) but during the wrong period). At 2500 milliseconds, the sound cue was presented, and participants were required to fixate on the TA (shaded area in e) as fast as possible. Fixation on the shaded area after 2500 milliseconds was defined as a target area during the proper period fixation (ie, saccade to the TA during the proper period).

To quantify the eye movements made during the different tasks, we divided the area viewed by participants into different areas (Figure 3): the TA, the SA, the center area (CA), the unrelated area (UA), the proper-side area (PSA), and the wrong-side area (WSA). The TA represented the area that participants were required to fixate on, and the SA represented the area of the stimulus. For the delayed saccade task, we further divided TA into TA during the proper period (TA-P) and TA during the wrong period (TA-W) to represent the TA area in the proper or wrong time periods, respectively (Figure 4). The TA and SA were the same in the prosaccade and delayed saccade tasks, whereas in the antisaccade task, they were horizontally symmetrical. The CA represented a 5 cm × 5 cm area around the central fixation cross. The UA was unrelated to the task requirements and expected to attract minimal attention during the tasks. The PSA and WSA were defined for the antisaccade task only and represented the proper and wrong areas, respectively, besides the CA.

### Extraction of Digital Biomarkers

#### Overview

On the basis of the eye-tracking paradigm, we calculated 28 digital biomarkers from the raw data recorded by the eye tracker. These biomarkers quantitatively reflect various behaviors of participants during the task, which were divided into 5 categories: general metrics (8/28, 29%), pupil-based metrics (4/28, 14%), area-based metrics (11/28, 39%), search-based metrics (3/28, 11%), and entropy-based metrics (2/28, 7%). For each assessment trial, we recorded 4 trial attributes (ie, task: prosaccades, antisaccades, and delayed saccades, target side: left and right, target eccentricity: 7°, 15°, and 20°, and trial order: first and second) and 6 participant attributes (ie, name, ID, category [ADHD and TD], sex [male and female], age, and age group). Table 1 summarizes these biomarkers in terms of category, symbol, description, and task.

**Table 1:** Descriptions of the digital biomarkers.

Category and symbol		Description	Task
**General metrics**			
	*N*_*Fix.*_	Total number of fixations	All^a^
	*N*_*Sac.*_	Total number of saccades	All
	*T*_*Total*_	Total duration of the trial	All
	*T*_*Fix. Avg.*_	Average fixation duration	All
	*T*_*Sac. Avg.*_	Average saccade duration	All
	*V*_*Sac. Avg.*_	Average saccade velocity	All
	*V*_*Sac. Peak*_	Peak value of saccade velocity	All
	*A*_*Sac. Avg.*_	Average saccade amplitude	All
**Pupil-based metrics**			
	*D*_*Pupil Avg.*_	Average pupil diameter	All
	*D*_*Pupil Max.*_	Maximum pupil diameter	All
	*D*_*Pupil Min.*_	Minimum pupil diameter	All
	*D*_*Pupil Sd.*_	SD of pupil diameter	All
**Area-based metrics**			
	*B*_*TA Fix.*_	Boolean value to signify the occurrence of fixations in the TA^b^ (TA-P^c^ for the delayed saccade task)	All
	*L*_*TA Fix.*_	Fixation latency of the TA (TA-P for the delayed saccade task)	All
	*N*_*UA Fix.*_	Number of fixations in the UA^d^	P^e^ and A^f^
	*N*_*TA Fix.*_	Number of fixations in the TA for the whole period	D^g^
	*N*_*TA-P Fix.*_	Number of fixations in the TA for the proper period	D
	*N*_*TA-W Fix.*_	Number of fixations in the TA for the wrong period	D
	*N*_*SA Fix.*_	Number of fixations in the SA^h^	A
	*B*_*PSA Fix.*_	Boolean value to signify the occurrence of fixations in the PSA^i^	A
	*B*_*WSA Fix.*_	Boolean value to signify the occurrence of fixations in the WSA^j^	A
	*B*_*PSA Fix. 1st*_	Boolean value to signify if the first fixation located in the PSA	A
	*B*_*Intrusive Sac.*_	Boolean value to signify the occurrence of intrusive saccade during the center fixation period	D
**Search-based metrics**			
	*B*_*Search*_	Boolean value to signify the occurrence of the search behavior	A
	*N*_*Search*_	Number of search behavior occurrences	A
	*T*_*Search*_	Total duration of search behavior	A
**Entropy-based metrics**			
	*SGE*_*norm*_	Normalized stationary gaze entropy	All
	*GTE*_*norm*_	Normalized gaze transition entropy	All

^a^All: all tasks, including prosaccade, antisaccade, and delayed saccade tasks.

^b^TA: target area.

^c^TA-P: target area during the proper period in the delayed saccade task.

^d^UA: unrelated area.

^e^P: prosaccade task.

^f^A: antisaccade task.

^g^D: delayed saccade task.

^h^SA: stimulus area.

^i^PSA: proper-side area.

^j^WSA: wrong-side area.

#### General Metrics

Human eye-movement patterns can be divided into fixations, saccades, and pursuits [36], of which the former 2 patterns are the focus of our paradigm. Using the Tobii Pro Lab software, we extracted the fixations and saccades of participants in chronological order from the raw gaze data. Subsequently, we calculated the total number of fixations (*N_Fix._*) and saccades (*N_Sac._*) and their average durations (*T_Fix. Avg._* and *T_Sac. Avg._*), which reflects participants’ holistic visual behavior. The velocity and amplitude of saccades were automatically recorded by the software. We calculated the average and peak saccade velocity (*V_Sac. Avg._* and *V_Sac. Peak_*) and the average saccade amplitude (*A_Sac. Avg._*) for each trial. These values reflect the scanning and information retrieval process, respectively. In addition, the total time taken for each trial (*T_Total_*) was recorded.

#### Pupil-Based Metrics

Pupil size is a crucial physiological measure that reflects autonomic nervous system activity, cognitive load, and emotional arousal. It has been applied extensively to various research fields [37-40]. The eye tracker continuously recorded participants’ pupil diameter during each trial. We preprocessed the raw data and extracted pupil-based metrics following 5 steps (Textbox 1) [41].

**Preprocessed raw data and extracted pupil-based metrics.**

Step 1: We removed samples labeled by the eye tracker as “invalid” and pupil diameters that fell outside the feasible range of 1.5 to 9.0 mm.

Step 2: We calculated pupil dilation speed to remove samples with a disproportionately large change in pupil size, which was usually caused by blinks or system errors. Because of the inconsistent sampling intervals, pupil diameter changes were not directly comparable between adjacent samples. Therefore, we calculated the normalized dilation speed between samples using the formula:

*s_i_* = max ( | (*p_i_* − *p_i_*_−1_) / (*t_i_* − *t_i_*_−1_) |, | (*p_i_*_+1_ − *p_i_*) / (*t_i_*_+1_ − *t_i_*) | ), **(1)**

where *p_i_* and *t_i_* are the pupil diameter sequence and timestamp sequence, respectively. To detect outliers in the dilation speed sequence (*s_i_*), we calculated the threshold, *T*, using the median absolute deviation (MAD):

*MAD* = median ( | *s_i_* – median ( *s_i_* ) | ), **(2)**

*T* = median ( *s_i_* ) + *n* ∙ *MAD*, **(3)**

where the scalar *n* was chosen as 1.5. Samples with an *s_i_* larger than *T* were removed as outliers. Because the eye tracker simultaneously collected data from both the left and right pupils, we performed steps 1 and 2 for each pupil separately.

Step 3: We excluded samples in which data of 1 pupil was missing and calculated the mean data sequence of the left and right pupil diameters.

Step 4: Because of nonuniform sampling and the presence of noise, we used a size 20 sliding window to resample and smooth the data sequence at 500 Hz. This involved an exponential moving average based on the timestamp and skipped data gaps ≥50 milliseconds.

Step 5: Following the above preprocessing steps, we obtained a valid, uniform, and smooth sequence of pupil diameter data. We then calculated the average (*D_Pupil Avg._*), maximum (*D_Pupil Max._*), minimum (*D_Pupil Min._*), and SD (*D_Pupil Sd._*) pupil diameter values of the sequence for each trial, which reflect various aspects of the pupil state of participants.

#### Area-Based Metrics

We extracted a range of metrics according to the area of interest (AOI) divisions. A Boolean value for fixation incidence (*B_TA Fix._*) was recorded to signify the completion of the task by detecting whether the TA (or TA-P for the delayed saccade task) contained any fixations. The latency of the first fixation in the TA (or TA-P) was recorded as the fixation latency (*L_TA Fix._*). The number of fixations was counted for the SA (only in the antisaccade task), UA (in the prosaccade and antisaccade tasks), TA-P (only in the delayed saccade task), and TA-W (only in the delayed saccade task), which were denoted as *N_SA Fix._*, *N_UA Fix._*, *N_TA-P Fix._*, and *N_TA-W Fix._*, respectively. For the delayed saccade task, fixations outside of the CA during the center fixation period were defined as intrusive saccades and thus recorded as a Boolean value (*B_Intrusive Sac._*). For the antisaccade task, if fixations were detected in the PSA (*B_PSA Fix._*) or WSA (*B_WSA Fix._*), these were recorded as Boolean values. We also used a Boolean metric to signify that the first fixation that occurred after the stimulus appeared was located in the PSA (*B_PSA Fix. 1st_*).

#### Search-Based Metrics

During the antisaccade task, participants may have had difficulty determining the correct fixation position, which may have led to a series of consecutive fixations around the TA before finally reaching the TA. In practice, we detected fixations in the surrounding area outside the TA and within a distance of 1.5 ∙ *L_TA_* from the TA center, where *L_TA_* is the length of the TA edge. Therefore, the consecutive sequences of ≥2 detected fixations were extracted as search behaviors. For each antisaccade trial, we recorded the following search-based metrics: the occurrence of search behaviors (*B_Search_*), the number of search behaviors (*N_Search_*), and their total duration (*T_Search_*).

Successful antisaccade trials required both a reversed saccade as well as an accurate landing position. Therefore, these metrics based on search behavior represent participants’ vision control and distance perception abilities.

#### Entropy-Based Metrics

Entropy in information theory [42] suggests that gaze entropy reflects the degree of uncertainty or predictability exhibited by the human eye during visual exploration. Thus, gaze entropy can provide valuable insight into the cognitive processes involved in visual perception and attention. There are 2 types of gaze entropy: stationary gaze entropy (SGE) and gaze transition entropy (GTE) [43]. SGE evaluates the spatial distribution of fixations, with a higher value indicating a more dispersed eye-movement pattern [44]. GTE focuses on the randomness of eye movements between fixations and reflects the flexibility and complexity of the scanning pattern.

**Figure 5:**
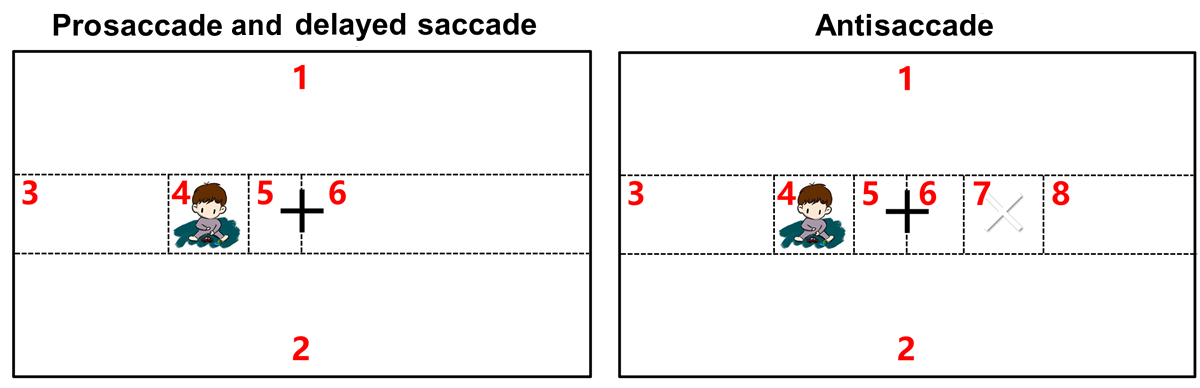
Division of areas for the calculation of gaze entropy metrics. It should be noted that the areas here are different from those for the area-based metrics shown in Figure 3.

As shown in Figure 5, the images were divided into *n* different areas, which served as the individual state spaces of a discrete system. We calculated the proportion of fixations located in each area, denoted as *p_i_* for the *i*-th area, which formed the approximate probability distribution of the states [45,46]. On the basis of the entropy equation by Shannon [42], SGE was calculated as follows:

*SGE* = – sum*_i_* ( *p_i_* ∙ log_2_
*p_i_* ). **(4)**

Applying the first-order Markov transition matrix [47], we derived *p*(*j*|*i*) from the fixation sequence, which represented the conditional probability of a gaze transitioning from the *i*-th to the *j*-th area. Then, GTE was computed based on the conditional entropy equation [47,48] as follows:

*GTE* = – sum*_i_* ( *p_i_* ∙ sum*_j_* ( *p*(*j*|*i*) ∙ log_2_
*p*(*j*|*i*) ) ). **(5)**

The maximum entropy of a system is determined by the number of available state spaces, which occurs when they are equally distributed [49]. To enable a comparison between different tasks, we used the corresponding maximum value, *H_max_* = log_2_ *n*, to normalize the computed SGE and GTE into a range from 0 to 1:

*SGE_norm_* = *SGE* / log_2_
*n*, **(6)**

*GTE_norm_* = *GTE* / log_2_
*n*. **(7)**

As introduced earlier, *n* represents the number of areas, where n=6 for the prosaccade and delayed saccade tasks, and n=8 for the antisaccade task.

#### Statistical Analysis

We reviewed and uniformly numbered basic information and scale data. After eliminating data with incomplete information, data were entered in duplicate using the Chinese version of EpiData 3.1 (The EpiData Association), and Excel (version 2019; Microsoft Corp) was used to clean and organize the data.

The Tobii Pro Lab software was used to analyze basic eye-movement metrics and export data. Participants with >80% valid data were included in the analysis. Python (version 3.8) was used to extract the eye-tracking metrics.

All data were tested for normality and homogeneity of variance. Samples conforming to a normal or approximately normal distribution are represented as means and SDs, and nonnormally distributed data are described as means and 95% CIs. Count data are expressed as n (%), and differences between groups were calculated using the chi-square test. For visual harmonization, 4 valid digits were retained for the eye-tracking metrics. We used independent samples 2-tailed *t* tests to compare normally distributed data between the 2 groups. To compare nonnormally distributed data between the 2 groups, we used the Wilcoxon Mann-Whitney *U* test, and the Kruskal-Wallis test was used to compare among multiple groups. Paired comparisons for significant multiple-group comparisons were performed using the Bonferroni method. A 2-sided *P<*.05 was considered statistically significant.

### ML Analysis

#### Overview

To validate the effectiveness of the proposed digital biomarkers, we conducted an ML analysis of the eye-tracking metrics to classify the ADHD and TD groups. First, we preprocessed the extracted metrics to meet the requirements of ML analysis and sequentially performed variable filtering, model construction, and model evaluation to verify the effectiveness of the extracted biomarkers. To ensure the reliability and generalizability of the model, we applied 5-fold cross-validation.

#### Data Preprocessing

The eye-tracking metrics were subdivided into multiple variables according to trial attributes (ie, task, target eccentricity, target side, and trial order). For each metric, we performed an average calculation for the target side and trial order, while maintaining different values for different task types and target eccentricities. For example, the metric *N_Fix._* was obtained from the prosaccade, antisaccade, and delayed saccade tasks with 7°, 15°, and 20° target eccentricities, respectively, which were subdivided into 9 variables as follows: *^P7^N_Fix._*, *^P15^N_Fix._*, *^P20^N_Fix._*, *^A7^N_Fix._*, *^A15^N_Fix._*, *^A20^N_Fix._*, *^D7^N_Fix._*, *^D15^N_Fix._*, and *^D20^N_Fix_*. This ensured that the variability of the metrics would be reasonably preserved. The preprocessing resulted in 183 eye-tracking variables, and each participant became 1 data point for the ML analysis.

#### Model Construction

Before model training, we performed filtering to remove redundant variables and enhance computational efficiency. Variables that were significantly different between groups, compared using the Mann-Whitney *U* test, were retained.

To predict the categories of participants, we used the extreme gradient boosting (XGBoost) algorithm as the classification model. XGBoost is an advanced implementation of the gradient boosting decision tree framework, which sequentially builds an ensemble of decision trees to refine the prediction. The learning process minimizes the gradient of the loss function, thereby enhancing the model’s performance. The XGBoost algorithm applies regularization techniques to efficiently boost the model and has thus demonstrated superior performance than the conventional gradient boosting decision tree framework in similar studies [50,51]. We implemented the XGBoost model in Python (version 3.8) using the packages *xgboost* (version 2.0.1) and *scikit-learn* (version 1.3.0). The hyperparameter settings of the model are listed in Multimedia Appendix 1, which are mainly the default values without adjustment to objectively illustrate the model’s performance.

#### Model Evaluation

The 5-fold cross-validation method with 500 repeats was applied to evaluate classification performance. The model was trained with 173 samples and tested with 43 samples for each fold. To evaluate the models, we used the receiver operating characteristic (ROC) curve and the area under the ROC curve (AUC), which consider the trade-off between the true positive rate and false positive rate at various classification thresholds and provide a holistic assessment of the model’s classification performance. We used the evaluation metrics of accuracy, sensitivity, specificity, precision, and *F*_1_-score to quantify classification performance.

#### Variable Importance

When training the XGBoost model, the split gain was calculated at each node of the decision tree, which indicated the contribution of variables to the model. After the training process, the split gain was aggregated for each variable among all the decision trees to provide a comprehensive measure of the variable’s relative importance in the classification of ADHD or TD groups.

## Results

### Characteristics of the Participants

A total of 216 participants (n=122, 56.5% in the TD group and n=94, 43.5% in the ADHD group) were enrolled in the study (Table 2). Overall, there was no significant difference in age (t_214_=–0.30; *P=*.76); full-scale IQ (t_214_=1.14; *P=*.25); or verbal IQ (t_214_=0.03; *P=*.98) between the TD and ADHD groups. However, the ADHD group scored significantly lower than the TD group for performance IQ (t_214_=2.08; *P=*.04). On the SNAP-IV, children in the TD group scored within the normal range, whereas the ADHD group scored significantly higher than the TD group on all 3 core symptoms (all *P<*.001).

**Table 2:** The basic information of the participants.

Variables		TD^a^ (n=122)	ADHD^b^ (n=94)	*t* test or chi-square test (*df*)^c^		*P* value	
**Sex, n (%)**					37.28 (1)		<.001
	Male	61 (50)	84 (89.4)				
	Female	61 (50)	10 (10.6)				
Age (y), mean (SD)		7.18 (1.19)	7.24 (1.39)	–0.30 (214)		.76	
**Age group, n (%)**					0.63 (2)		.73
	Group 1 (5-6 y)	44 (36.1)	36 (38.3)				
	Group 2 (7-8 y)	45 (36.9)	37 (39.4)				
	Group 3 (9-10 y)	33 (27)	21 (22.3)				
**IQ, mean (SD)**							
	Verbal IQ	97.36 (12.51)	97.41 (12.90)	0.03 (214)		.98	
	Performance IQ	103.02 (13.25)	99.01 (15.02)	2.08 (214)		.04	
	Full-scale IQ	100.06 (12.44)	98.11 (12.43)	1.14 (214)		.25	
**SNAP-IV** ^d^ **, mean (SD)**							
	Inattentive	0.63 (0.25)	15.09 (0.75)	–199.20 (214)		<.001	
	Hyperactivity or impulsive	0.50 (0.30)	11.59 (0.82)	–137.43 (214)		<.001	
	Oppositional defiant	0.36 (0.12)	7.66 (0.56)	–118.54 (214)		<.001	

^a^TD: typically developing.

^b^ADHD: attention-deficit/hyperactivity disorder.

^c^*t*-tests were used for variables presenting means and standard deviations (Age, IQ, and SNAP-IV scores), and chi-square tests were used for variables presenting numbers and percentages (Sex and Age group).

^d^SNAP-IV: Swanson, Nolan, and Pelham Rating Scale.

### Comparison of Digital Biomarkers Between the ADHD and TD Groups

#### Eye-Tracking Metrics Across the 3 Tasks

**Figure 6:**
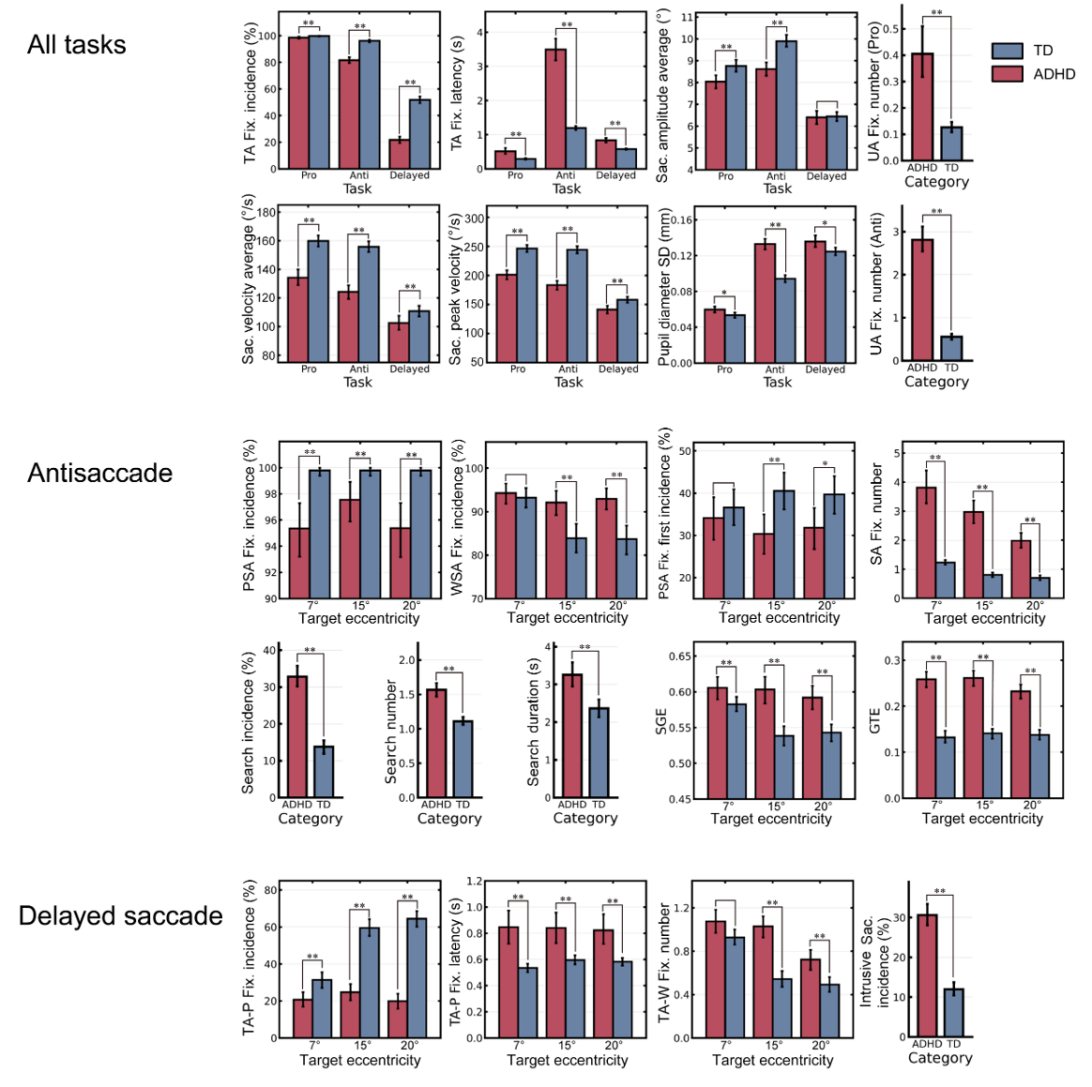
Comparisons of eye-tracking metrics between the attention-deficit/hyperactivity disorder (ADHD) and typically developing (TD) groups. Results of the corresponding data analyses are presented in Multimedia Appendices 3 and 4. *P<.05, **P<.01. Fix.; fixation; GTE: gaze transition entropy; PSA: proper-side area; SA: stimulus area; Sac.: saccade; SGE: stationary gaze entropy; TA: target area; UA: unrelated area; WSA: wrong-side area.

The analysis of the biomarkers identified for all 3 tasks (Figure 6; Multimedia Appendices 2 and 3) showed that for completion, there were significant differences in TA fixation incidence (calculated based on *B_TA Fix._*) and *L_TA Fix._* between the ADHD and TD groups for all 3 tasks (both *P<*.001). *A_Sac. Avg._* of the ADHD group was significantly smaller than that of the TD group in the prosaccade and antisaccade tasks (all *P<*.001), whereas *V_Sac. Avg_* and *V_Sac. Peak_* of the ADHD group was significantly slower than those of the TD group for all tasks (all *P<*.001). *D_Pupil Sd._* of the ADHD group was significantly greater than that of the TD group for all tasks (*P=*.03 for the prosaccade task, *P<*.001 for the antisaccade task, and *P=*.02 for the delayed saccade task).

In terms of attention control, in both the prosaccade and antisaccade tasks, more irrelevant fixations (ie, *N_UA Fix._*) occurred in the ADHD group than in the TD group (all *P<*.001). In addition, the ADHD group fixated more frequently on the UA during the antisaccade task than in the prosaccade task.

#### Eye-Tracking Metrics of the Antisaccade Task

**Figure 7:**
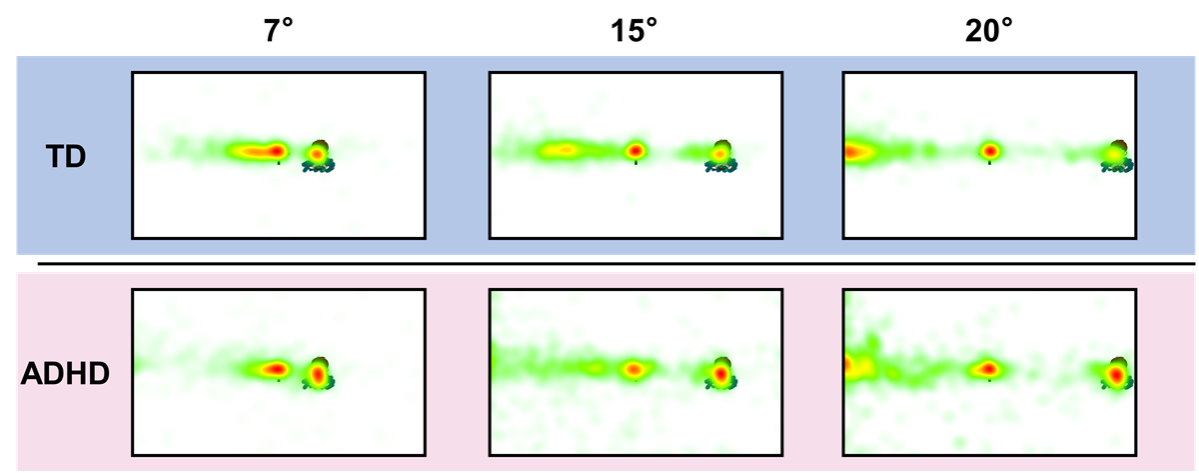
Heat maps of fixations of the typically developing (TD) and attention-deficit/hyperactivity disorder (ADHD) groups for stimuli of different target eccentricities in the antisaccade task.

The heat maps (Figure 7) of the analysis of the different target eccentricities (Multimedia Appendix 4) revealed that the TD group’s fixations were concentrated along the horizontal position where the SA and TA were located, whereas the ADHD group’s fixations were more widespread. Moreover, the TD group was more accurate than the ADHD group in fixating on the TA, whereas the ADHD group showed more erroneous localization deviations in both the 7° and 15° trials. Interestingly, in the 20° trial, we noted that the fixation concentration of the ADHD group deviated from the stimulus: there was a longitudinal distribution of fixations along the edge of the correct side of the screen, which suggested that the ADHD group did not localize fixation according to the logic of symmetry; rather, they relied purely on the edge of the screen to assist in their fixation positioning.

As shown in Figure 6 and Multimedia Appendix 4, the ADHD group had more WSA fixations (calculated from *B_WSA Fix._*) and fewer PSA fixations (calculated from *B_PSA Fix._*) than the TD group (all *P<*.001). Among the 3 eccentricities, the number of WSA fixations during the 15° and 20° trials were significantly different between the groups (*U*=81,316 for 15°, *U*=80,812 for 20°, all *P<*.001), whereas in the 7° trials, both groups showed a higher number of WSA fixations (*U*=87,841, *P=*.52) than PSA fixations. However, the TD group had more PSA fixations in the 7° trials and a higher incidence of the first fixation in the PSA (calculated from *B_PSA Fix. 1st_*) than the ADHD group (all *P<*.001).

Comparisons of search incidence (calculated from *B_Search_*), *N_Search_*, and *T_Search_* between the ADHD and TD groups showed that the ADHD group was significantly higher than the TD group for all 3 metrics (*P<*.001, *P<*.001, and *P=*.008, respectively). Both SGE and GTE were significantly higher in the ADHD group than in the TD group (all *P<*.001).

#### Eye-Tracking Metrics in the Delayed Saccade Task

As shown in Figure 6 and Multimedia Appendix 4, TA-P fixation incidence (calculated from *B_TA Fix._*) and *L_TA Fix._* were significantly different between the 2 groups at all eccentricities. Moreover, the TD group had a lower *N_TA-W Fix._* than the ADHD group (all *P<*.001).

As the stimulus eccentricity increased from the center point, only the TD group showed an improvement in performance. The TD group showed a lower *N_TA-W Fix_* when the eccentricity was 15° than when the eccentricity was 7°, whereas the decrease in *N_TA-W Fix_* in the ADHD group from an eccentricity of 15° to 20° was more gradual than that in the TD group.

The assessment of intrusive saccades for stability of eye movements showed that the ADHD group had more intrusive saccades (calculated from *B_Intrusive Sac._*) and less stable eye-movement patterns than the TD group (*P<*.001).

### Comparisons of Digital Biomarkers Among Age Groups

**Figure 8:**
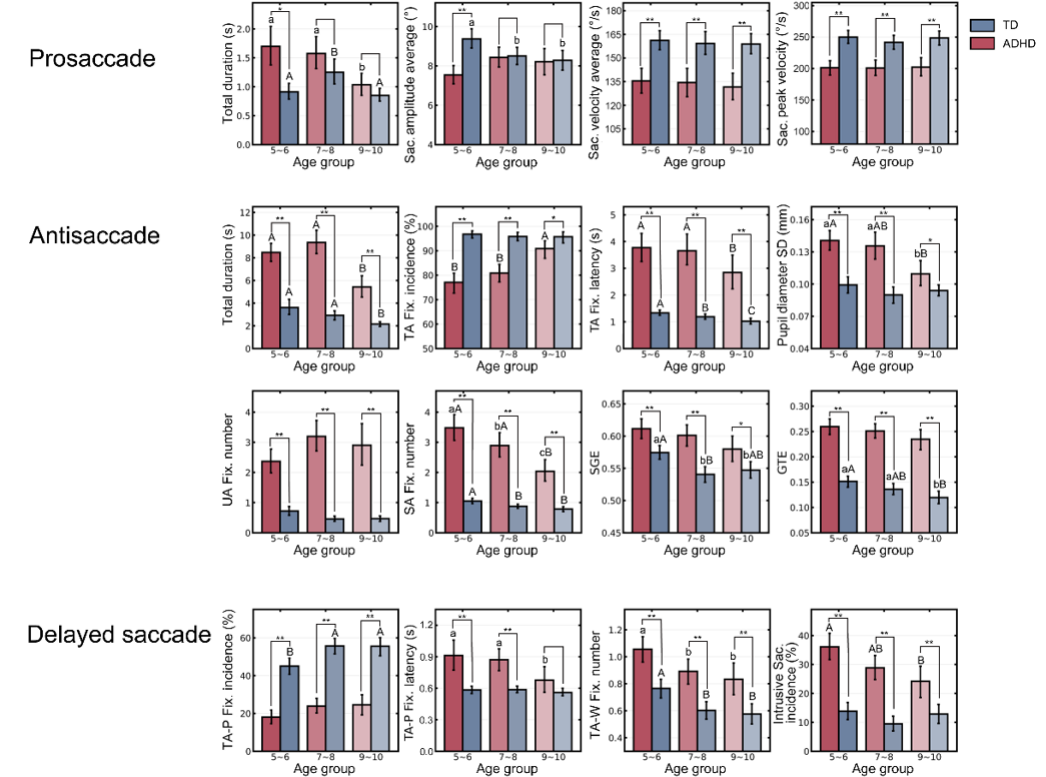
Comparisons of eye-tracking metrics among age groups. Letters above the bars indicate the results of the post hoc tests using Bonferroni correction among different age groups in the attention-deficit/hyperactivity disorder (ADHD) and typically developing (TD) groups. Lower case letters indicate *P*<.05; upper case letters indicate *P*<.01. *P<.05, **P<.01. Fix.; fixation; GTE: gaze transition entropy; SA: stimulus area; Sac.: saccade; SGE: stationary gaze entropy; TA: target area; UA: unrelated area.

We discovered that several digital biomarkers showed consistent changes with age (Figure 8; Multimedia Appendices 5 and 6). In the prosaccade task, the overall *T_Total_* of both groups showed a decreasing trend with age (*P=*.02 for ADHD, *P<*.001 for TD). In addition, an age-related decrease in *A_Sac. Avg._* was observed in the TD group only (*P=*.007), whereas *V_Sac. Avg._* and *V_Sac. Peak_* remained stable in both groups (*P=*.71 for *V_Sac. Avg._* and *P*=.46 for *V_Sac. Peak_*). In the antisaccade task, both the TD and ADHD groups showed an increasing trend for accuracy (*P<*.001 for ADHD, *P=*.63 for TD) and efficiency (*P<*.001 for ADHD and TD) in completing the task. In fact, the ADHD group showed significantly greater improvement than the TD group (*P<*.001). The ADHD group also exhibited a propensity for *D_Pupil Sd._* to decrease with age (*P<*.001). Across all age groups, the ADHD group had a higher *N_UA Fix._* than the TD group (*P<*.001), and this did not significantly improve with age; although the *N_SA Fix._* significantly dropped with age (*P<*.001). We also found that there was a greater tendency for SGE and GTE to decline with age in the TD group than in the ADHD group (*P<*.001 for SGE and *P=*.001 for GTE).

The TA-P fixation incidence (*P=*.06) did not significantly differ with age in the ADHD group for the delayed saccade task. This was true despite the ADHD group showing improvements in *L_TA Fix._* (*P=*.01), *N_TA-W Fix._* (*P=*.005), and intrusive saccade incidence (calculated from *B_Intrusive Sac._*; *P=*.003) with age.

### ML Analysis With the Proposed Digital Biomarkers

**Figure 9:**
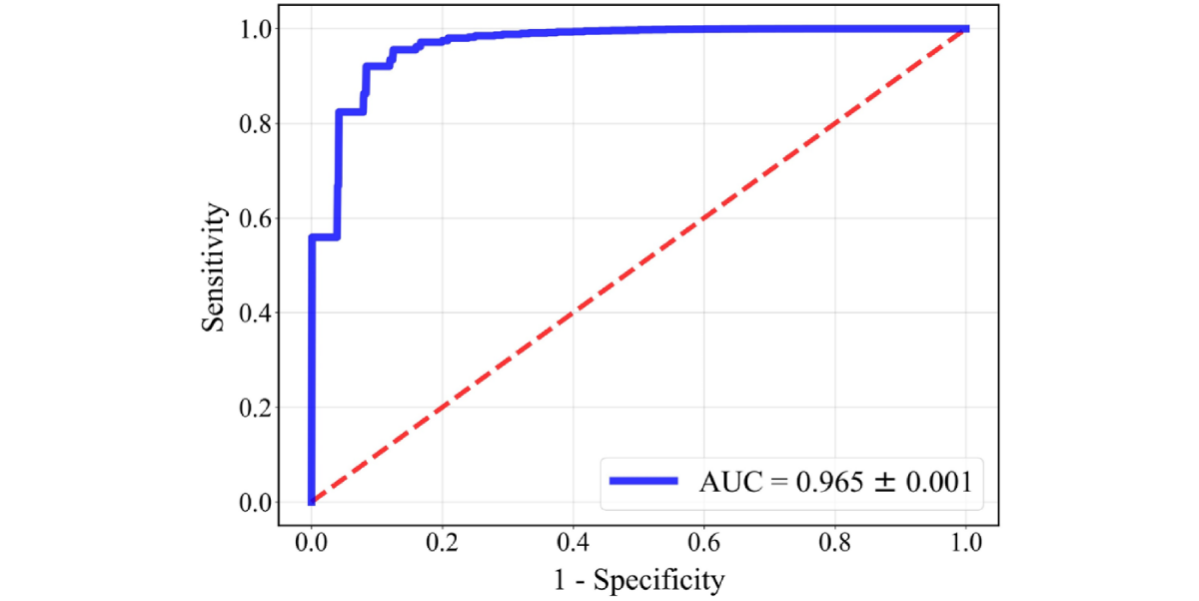
Receiver operating characteristic curve of the classification model. AUC: area under the receiver operating characteristic curve.

**Figure 10:**
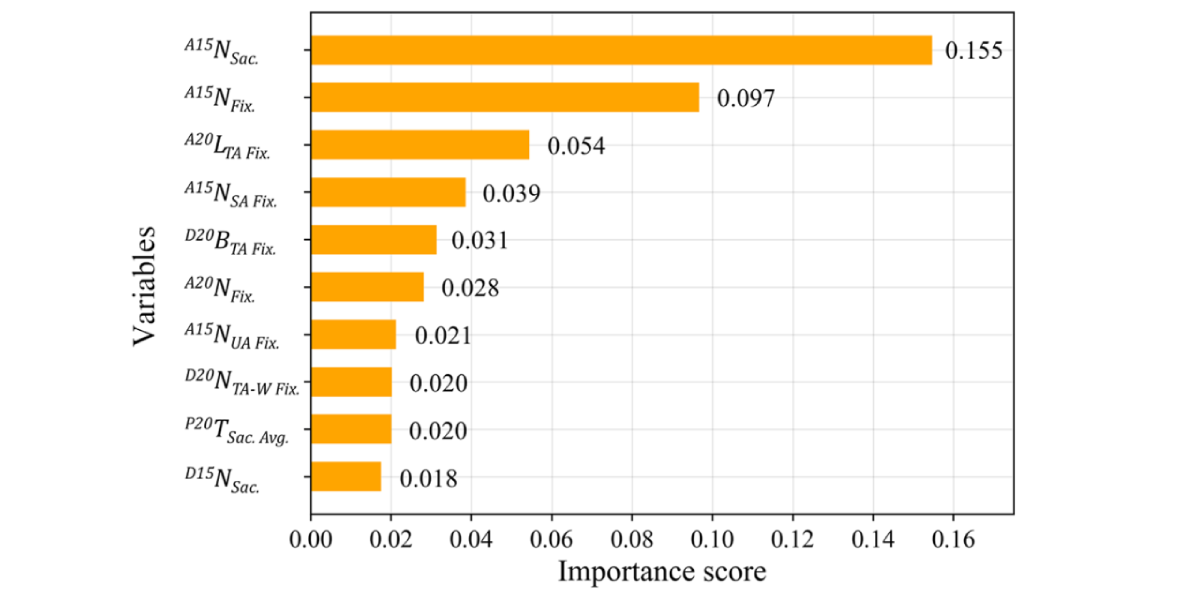
Importance scores of the top 10 most important variables. N_Sac._: total number of saccades; N_Fix._: total number of fixations; L_TA Fix._: fixation latency of the TA; N_SA Fix._: number of fixations in the stimulus area; B_TA Fix._: Boolean value to signify the occurrence of fixations in the TA (TA-P for the delayed-saccade task); N_UA Fix._: number of fixations in the UA; N_TA-W Fix._: number of fixations in the TA for the wrong period; T_Sac. Avg._: average of saccade duration.

The evaluation metrics (AUC, accuracy, sensitivity, specificity, precision, and *F*_1_-score) are reported as means (95% CIs). The XGBoost model trained on the eye-tracking variables achieved an AUC of 0.965 (0.964-0.966), an accuracy of 0.908 (0.907-0.910), a sensitivity of 0.877 (0.874-0.880), a specificity of 0.932 (0.930-0.934), a precision of 0.913 (0.910-0.915), and an *F*_1_-score of 0.892 (0.890-0.894). The averaged ROC curve is shown in Figure 9, which illustrates the effectiveness of the proposed digital biomarkers for discriminating the ADHD and TD groups. The 10 most important variables for the model are reported with their scores in Figure 10.

## Discussion

### Principal Findings

#### Clinical Behavioral Performance

The 3 saccade tasks consistently showed that the performance of the ADHD group was poorer than that of the TD group, which suggests that the paradigm serves as a reliable and objective measure of cognitive and executive functioning. Furthermore, the ADHD group exhibited a pattern of amelioration with aging, whereas the TD group showed consistent performance across the different age groups. This may be because TD individuals had already achieved a higher cognitive skill level and a relatively stable state of corresponding biomarkers than ADHD individuals of the same age. Therefore, despite the ADHD group showing a faster rate of improvement, they performed significantly worse than TD individuals across all age groups. This finding demonstrates distinct developmental eye-movement patterns associated with ADHD.

#### Attention and Inhibitory Control

The ADHD group exhibited a significant lag in the ability to inhibit stimuli, which was characterized by poorer performance than the TD group on tasks with a weaker perceptual load. Previous studies have confirmed that human visual features are divided into 3 regions–the foveal region at a viewing angle of 2.5° from the gaze point has the highest visual sensitivity, followed by the parafoveal region from 2.5° to 4.2°, and the peripheral region from 4.2° to 9.2° has the lowest visual sensitivity [52]. In this study, the 7° eccentricity stimulus was closest to the central cross and within the peripheral region, whereas the other 2 stimulus types were located outside the peripheral region. Thus, the task of inhibiting the 15° and 20° eccentricity stimuli was a low perceptual load task, which was relatively easy for the TD group. However, the performance of the PSA first incidence showed that the ADHD group had poorer inhibitory control for the lower perceptual load task of inhibiting stimuli that were located outside of the peripheral region (ie, the 15° and 20° eccentricity stimuli, as shown in Figure 6). This confirms the existence of up-down attention control impairment in individuals with ADHD [53] and emphasizes that children with ADHD may be more prone to distraction in low perceptual load environments because of a higher central threshold of response to perceptual load [54]. This finding also corroborates previous reports that individuals with ADHD are more sensitive to stimuli located in peripheral regions.

Furthermore, although individuals with ADHD had difficulty suppressing the sudden appearance of distracting stimuli, they also had a longer completion time than the TD group for the prosaccade task with a single instruction. This may be attributed to the low load of the prosaccade task, which may not have elicited sufficient cognitive arousal in the ADHD group, leading to poorer task performance. In addition, in the delayed saccade tasks that involved sequential instructions (ie, “do not look at the stimulus until you hear the cue, and then quickly look at the stimulus”), the weak task-switching ability of the ADHD group may have also prolonged fixation latency.

#### Organizing and Planning

In the antisaccade task, the ADHD group exhibited significantly lower TA fixation incidence and longer L*_TA Fix._* compared with the TD group (Figure 6). This suggests that most children in the ADHD group were unable to accurately localize the TA, and those who succeeded took longer. On the basis of the heat map and UA fixation (Figures 6 and 7), the ADHD group exhibited greater fixation deviation and more frequent search behaviors.

In addition, the ADHD group had much higher SGE and GTE than the TD group for overall eye-movement trajectory, which indicated that they exhibited more eye-movement pattern shifts and spatial dispersion of fixations. This suggests that patients with ADHD favor an irregular search pattern and lack forethought when organizing and coordinating eye movements during symmetrical localization, resulting in prolonged search time to accurately locate the target. Furthermore, the positive correlation between SGE and GTE in the ADHD group supports the impact of top-down interference on visual scanning in ADHD [43].

The TD group followed a significant declining trend in SGE (*P<*.001) and GTE (*P=*.001) with age, whereas the ADHD group maintained high entropy values. We also observed that the frequent UA fixation in the ADHD group did not improve with age. These findings suggest that with age, the TD group better localized the landing point, which led to a more regular eye-movement trajectory. In contrast, the irregular eye-movement pattern of the ADHD group was exhibited across all age groups.

#### Eye-Movement Coordination With Age

Previous studies have mainly focused on age-related changes in the general population by comparing individuals among different age groups. However, few studies have examined variations in eye movement among younger individuals with ADHD and TD individuals. A recent study evaluating the performance of visually guided horizontal prosaccades in healthy people aged 3 years to >80 years found that peak saccade velocity increases until the age of 6 years, after which it remains relatively stable until 10 years of age [55]. The results of our prosaccade task similarly demonstrated that *V_Sac. Avg._* and *V_Sac. Peak_* remained stable from ages 5 to 10 years in both the ADHD and TD groups, which indicates that the developmental pattern of saccade velocity is similar across both groups.

We also discovered that the ADHD group was more likely to experience intrusive saccades during the central fixation stage. The percentage of intrusive saccades decreased with age in the ADHD group, whereas that in the TD group remained at a well-performing and stable level across age groups. This further highlights the overall impairment in eye-movement control in the ADHD group.

The TD group showed a consistently higher *V_Sac. Avg._* than the ADHD group. However, it showed a decreasing trend with age for A*_Sac. Avg_* than the ADHD group. In addition to speed, accurate localization is also required to successfully perform the prosaccade task. With age, children may modulate their eye movements to a lower speed for greater controllability, rather than simply sweeping their eyes rapidly toward the target, and thus, increase task efficiency.

Previous research has reported that the cerebellum is a crucial hub of the motor network that interacts with the executive control circuits of the frontoparietal lobe, which are involved in inhibition and stimulus response [56]. Furthermore, studies have demonstrated reduced volume and under activation of the cerebellum in individuals with ADHD [57], which suggests that impairment of the cerebellum contributes to poor control and coordination of eye movements in patients with ADHD.

#### Variations in Pupil Diameter and Cognitive Stress

It is well-established that when humans encounter stressful situations, they dilate their pupils to improve vision [58]. Previous research using eye-tracking technology has also revealed that when people are engaged in an active coping task, their pupils enlarge significantly. These findings suggest that a larger pupil diameter is linked to higher cognitive load while preparing for challenging tasks [58]. According to previous research examining the relationship between pupil diameter and attention, there is an inverted U–shaped pattern between pupil diameter and attentional performance; that is, when pupil diameter becomes too small or large, error rates are higher and response times are slower [59]. In our study, we discovered that for all tasks, children with ADHD displayed greater pupil diameter variation than TD individuals. This finding supports the theory that excessively large or small pupil diameter is an indicator of inattentiveness when completing tasks requiring active responses. Alternatively, executive function deficiencies at the functional level of the brain and inefficient brain network connectivity in the ADHD group may account for the higher cognitive load when responding to complex task demands [60].

#### ML Analysis

For the classification of ADHD and TD children, the ML model achieved an AUC of 0.965 and an accuracy of 0.908, which demonstrates promise for the model to serve as an automated screening tool for ADHD children. Moreover, the high performance of the model highlights the effectiveness of the paradigm and its ability to extract digital eye-tracking biomarkers. In a previous study focused on screening for ADHD using eye-tracking and ML methods, Lev et al [18] conducted continuous performance tests in 66 participants (33 adult patients with ADHD and 33 healthy controls) and used eye-movement metrics during the tests to classify patients and controls. They applied a regression model to combine the relative gaze durations of 4 AOIs as the diagnostic scale and achieved an AUC of 0.826. Das and Khanna [19] extracted pupil size dynamics features as an objective biomarker and trained 5 types of commonly used classification models to detect ADHD. Using the data of 50 participants (28 patients with ADHD and 22 healthy controls) and 10-fold cross-validation, they attained an AUC of 0.856. Deng et al [61] built an eye-tracking ML classifier for ADHD using the natural reading paradigm; however, the model was difficult to interpret, and the classification performance (AUC of 0.646) was not as high as the performance achieved by our model.

Compared with previous work, we recruited a larger number of participants (ie, 94 ADHD and 122 TD individuals), obtained higher evaluation metrics, and achieved better classification performance for children with ADHD. Moreover, we extracted a larger variety of eye-tracking metrics and provided a more comprehensive description of participants’ eye-movement behaviors. These advantages emphasize the effectiveness, reliability, and potential practical applications of the model. Furthermore, our findings offer valuable insight into the field of ADHD diagnosis using ML.

Because we plan to extend our findings using portable eye-tracking devices in the future, we validated the performance of our model at lower sampling frequencies using external samples. Results demonstrated that the model adapted well to low-sampling rate data, which further confirmed its high generalizability and applicability to portable devices (Multimedia Appendix 7).

### Advantages of the Study

First, we used eye-tracking technology in a natural and straightforward assessment setting, which enabled direct visual and on-screen interactions without complicated rules or restrictions on head motion. Unlike the paradigms used in previous studies, our approach did not require participants to wear additional equipment [7] or make additional keystrokes [18]. In addition, our method avoided interference from other environments and devices, facilitated children’s participation, and minimized inaccuracies in eye-movement measurement due to excessive head movement.

Second, our paradigm allowed a more comprehensive exploration of children’s cognitive skills. In addition to testing attentional and inhibitory ability, our paradigm included audiovisual integration, which has been shown to be effective in evaluating children with ADHD.

Third, we provided a more comprehensive scheme for extracting digital eye-tracking biomarkers by expanding the evaluation system of classical paradigms. The presentation of stimuli was further divided into defined areas of fixation for quantitative analyses; moreover, behaviors, such as search behaviors that are typically observed in the clinic, were quantified alongside numerous metrics based on the AOI, such as fixation duration, saccade velocity and amplitude, and pupil diameter change. This enabled the extraction of more detailed eye-movement metrics during different saccade tests than those used in previous studies [8,18,19,62] while ensuring that the extracted digital biomarkers were interpretable and objectively reflected cognitive deficits. As a result, we were able to provide a practical and thorough description of children’s performance in completing the various tasks.

In addition, we applied ML modeling using the extracted digital biomarkers and achieved promising results, which confirmed that these biomarkers are highly valuable for the future development of screening and auxiliary diagnostic tools. We also investigated age-related developmental patterns of eye movement in addition to simple eye-movement metrics in children with ADHD in a larger, more trustworthy, and more representative dataset than previous research. In terms of practical applications, the implementation of the paradigm is straightforward, and the 7-minute duration of the assessment is suitable for children with ADHD. These features will increase the likelihood that the assessment can be completed successfully by children with ADHD. Taken together, we have provided a reliable and practical solution for auxiliary diagnosis and screening for ADHD at the primary care level.

### Limitations

Although our sample size was larger than previous studies, we only recruited from 1 city in China. Therefore, the representativeness of the sample can be improved. There was also a sex imbalance between the TD and ADHD groups. The TD group had a 1:1 sex ratio, whereas the ADHD group had considerably more boys (84/94, 89%) than girls (10/94, 11%). Although this was attributed to morbidity bias, our modeling would have benefited from a balanced sex ratio, especially because previous studies have reported sex differences in comorbidity and cognitive impairment in children with ADHD [63]. To verify the effect of sex on the validity of the model, 2 models were trained using data from boys and girls separately and validated using data from the opposite sex. Results showed that the 2 models performed well, which suggested that sex does not significantly affect modeling and that the current model trained with predominantly male data can also be applied to predict female participants’ behavior (Multimedia Appendix 8). Nevertheless, we must consider recruiting participants with various presentations (eg, different geography and balanced sex ratios) in future studies to ensure the generalizability of the ML model. Finally, our eye-tracking instrument is heavy and difficult to carry. Thus, in the future, a portable eye-tracking instrument could be used.

### Conclusions and Future Research

We successfully adapted eye-tracking technology for clinical use as a tool for auxiliary diagnosis and campus and community screening for ADHD. The system includes standard paradigms and a reliable digital biomarker extraction process. We validated the use of digital biomarkers to build robust ML models. In addition, the entire assessment process was conducted in a natural setting without the need for extra equipment to be worn by participants. The assessment is also brief and simple, which makes it particularly suitable for clinical applications and ensures completion of the assessment.

For the next steps of our research, we plan to further expand the sample size and implement multicenter data collection using the proposed paradigm and digital biomarker extraction scheme. We aim to build a robust ML model and externally validate classifiers to improve their predictive accuracy and stability. This will ensure that the auxiliary diagnosis model can be effectively applied to real clinical scenarios and improve primary care–level screening and diagnosis of ADHD.

## Appendix

Multimedia Appendix 1 Hyperparameters of XGBoost model.

Multimedia Appendix 2 Detailed description of digital biomarkers illustrated in result figures.

Multimedia Appendix 3 Differences in eye-movement metrics between attention-deficit/hyperactivity disorder and typically developing groups.

Multimedia Appendix 4 Differences in eye-movement metrics between attention-deficit/hyperactivity disorder and typically developing groups in the antisaccade and delayed saccade tasks with different target eccentricities.

Multimedia Appendix 5 Differences in eye-movement metrics between attention-deficit/hyperactivity disorder and typically developing groups for different age groups.

Multimedia Appendix 6 Differences in eye-movement metrics between different age groups for attention-deficit/hyperactivity disorder and typically developing groups.

Multimedia Appendix 7 Validation of the impact of down-sampling on model training.

Multimedia Appendix 8 Validation of the effect of sex differences on model training.

## Abbreviations

ADHD: attention-deficit/hyperactivity disorder
AOI: area of interest
AUC: area under the receiver operating characteristic curve
CA: center area
GTE: gaze transition entropy
ML: machine learning
PSA: proper-side area
ROC: receiver operating characteristic
SA: stimulus area
SGE: stationary gaze entropy
SNAP-IV: Swanson, Nolan, and Pelham Rating Scale
TA: target area
TA-P: target area during the proper period
TA-W: target area during the wrong period
TD: typically developing
UA: unrelated area
WSA: wrong-side area
XGBoost: extreme gradient boosting
