# Supplementary material for: Auxiliary Diagnosis of Children With Attention-Deficit/Hyperactivity Disorder Using Eye-Tracking and Digital Biomarkers: Case-Control Study
Source: JMIR Mhealth Uhealth. 2024 Nov 29;12:e58927. doi: 10.2196/58927 (PMC11645504; doi:10.2196/58927)
Supplement: Multimedia Appendix 1 [file mhealth_v12i1e58927_app1.docx]

**Appendix 1. Hyper-parameters of XGBoost model.**

| **Hyper-parameter** | **Value** |
| --- | --- |
| Learning rate | 0.1 |
| L1 regularization | 0.05 |
| L2 regularization | 0.05 |
| Estimator number | 100 |
| Maximum depth | 6 |
| Subsample ratio | 1 |
| Minimum child weight | 1 |
| Minimum split loss | 0 |
| Importance type | Gain |
