# Supplementary material for: Auxiliary Diagnosis of Children With Attention-Deficit/Hyperactivity Disorder Using Eye-Tracking and Digital Biomarkers: Case-Control Study
Source: JMIR Mhealth Uhealth. 2024 Nov 29;12:e58927. doi: 10.2196/58927 (PMC11645504; doi:10.2196/58927)
Supplement: Multimedia Appendix 2 [file mhealth_v12i1e58927_app2.docx]

**Appendix 2. Detailed description of digital biomarkers illustrated in result figures.**

| Task | Symbol | Digital biomarkers | Detailed description |
| --- | --- | --- | --- |
| All tasks | *B_TA Fix._* | TA Fix. incidence | The occurrence (incidence) of fixation in the TA (during the proper period for delayed saccade task), indicating the ratio of successfully completing tasks. |
|  | *L_TA Fix._* | TA Fix. latency | The latency between the appearance of stimulus and the first fixation in the TA (during the proper period for delayed saccade task), indicating the reaction time required to complete tasks. |
|  | *A_Sac. Avg._* | Sac. amplitude average | The average value of saccade amplitude. |
|  | *N_UA Fix._* | UA Fix. number (Pro / Anti) | The number of fixations in the UA, partially reflecting the level of distraction. |
|  | *V_Sac. Avg._* | Sac. velocity average | The average value of saccade velocity. |
|  | *V_Sac. Peak_* | Sac. peak velocity | The peak velocity of saccades, indicating the maximum value of saccade velocity. |
|  | *D_Pupil Sd._* | Pupil diameter SD | The standard deviation of pupil diameter, indicating the degree of variation in pupil diameter. |
| Prosaccade | *T_Total_* | Total duration | The total duration of completing tasks. |
|  | *A_Sac. Avg._* | Sac. amplitude average | The average value of saccade amplitude. |
|  | *V_Sac. Avg._* | Sac. velocity average | The average value of saccade velocity. |
|  | *V_Sac. Peak_* | Sac. peak velocity | The peak velocity of saccades, indicating the maximum value of saccade velocity. |
| Antisaccade | *B_PSA Fix._* | PSA Fix. incidence | The occurrence (incidence) of fixation in the PSA, indicating the ratio of directional accuracy. |
|  | *B_WSA Fix._* | WSA Fix. incidence | The occurrence (incidence) of fixation in the WSA, indicating the ratio of directional error. |
|  | *B_PSA Fix. 1st_* | PSA Fix. first incidence | The occurrence (incidence) of the first fixation located in the PSA, indicating the ability to locate the target efficiently. |
|  | *N_SA Fix._* | SA Fix. number | The number of fixations in the SA, indicating the degree of directional error. |
|  | *B_Search_* | Search incidence | The occurrence (incidence) of search behavior. |
|  | *N_Search_* | Search number | The number of search behaviors. |
|  | *T_Search_* | Search duration | The total duration of search behavior in one trial. |
|  | *SGE_norm_* | SGE | The normalized value of stationary gaze entropy (SGE). |
|  | *GTE_norm_* | GTE | The normalized value of gaze transition entropy (GTE). |
|  | *T_Total_* | Total duration | The total duration of completing tasks. |
|  | *B_TA Fix._* | TA Fix. incidence | The occurrence (incidence) of fixation in the TA, indicating the ratio of successfully completing tasks. |
|  | *L_TA Fix._* | TA Fix. latency | The latency between the appearance of stimulus and the first fixation in the TA, indicating the reaction time required to complete tasks. |
|  | *D_Pupil Sd._* | Pupil diameter SD | The standard deviation of pupil diameter, indicating the degree of variation in pupil diameter. |
|  | *N_UA Fix._* | UA Fix. number | The number of fixations in the UA, partially reflecting the level of distraction. |
| Delayed saccade | *B_TA Fix._* | TA-P Fix. incidence | The occurrence (incidence) of fixation in the TA during the proper period, indicating the ratio of successfully completing tasks. |
|  | *L_TA Fix._* | TA-P Fix. latency | The latency between the appearance of stimulus and the first fixation in the TA during the proper period, indicating the reaction time required to complete tasks. |
|  | *N_TA-W Fix._* | TA-W Fix. number | The number of fixations in the TA during the wrong period. |
|  | *B_Intrusive Sac._* | Intrusive Sac. incidence | The occurrence (incidence) of intrusive saccades during the center fixation period. |
