# Supplementary material for: Auxiliary Diagnosis of Children With Attention-Deficit/Hyperactivity Disorder Using Eye-Tracking and Digital Biomarkers: Case-Control Study
Source: JMIR Mhealth Uhealth. 2024 Nov 29;12:e58927. doi: 10.2196/58927 (PMC11645504; doi:10.2196/58927)
Supplement: Multimedia Appendix 3 [file mhealth_v12i1e58927_app3.docx]

**Appendix 3. Differences in eye movement metrics between ADHD and TD groups.**

Note: MAX for the maximum of such eye movement characterization metrics at this task, MIN for the minimum of such eye movement characterization metrics at this task, SD for the standard deviation.

| **Task types** | **Digital biomarkers** | **ADHD Mean(95%CI)** | **TD Mean(95%CI)** | **Confidence interval for median differences Difference (95%CI for Difference)** | ***U*** | ***P*** |
| --- | --- | --- | --- | --- | --- | --- |
| **Prosaccade** | Total duration (ms) | 1527.0(1367.0~1687.0) | 960.5(899.5~1022.0) | -34.000(-66.000~-17.000) | 264753 | <.001 |
|  | TA Fix. incidence | 0.9858(0.9789~0.9927) | 0.9973(0.9946~0.9999) | 4.671e-05(-4.702e-05~4.668e-05) | 835152 | <.001 |
|  | TA Fix. latency (ms) | 514.9(425.3~604.5) | 289.4(265.0~313.9) | -14.000(-21.000~-6.000) | 744148 | <.001 |
|  | Fix. number | 4.658(4.318~4.999) | 3.262(3.157~3.368) | -2.663e-05(-6.797e-05~-4.869e-05) | 684394 | <.001 |
|  | Fix. duration average (ms) | 200.0(194.2~205.9) | 204.7(199.4~210.0) | 5.000(0.667~9.333) | 864819 | .024 |
|  | Sac. number | 7.826(6.882~8.77) | 4.023(3.46~4.585) | -1.000(-1.000~-1.000) | 580766 | <.001 |
|  | Sac. duration average (ms) | 32.67(31.86~33.48) | 38.15(37.44~38.86) | 5.917(4.833~7.000) | 1015526 | <.001 |
|  | Sac. velocity average (°/s) | 134.2(129.1, 139.3) | 159.8(156.1, 163.6) | 30.180(24.865~35.520) | 1027223 | <.001 |
|  | Sac. peak velocity (°/s) | 201.3(193.7, 208.9) | 246.5(240.3, 252.7) | 50.88(41.700~60.055) | 1023716 | <.001 |
|  | Sac. amplitude average (°) | 8.035(7.745~8.325) | 8.757(8.502~9.011) | 0.730(0.363~1.105) | 750839 | <.001 |
|  | Pupil diameter SD (mm) | 0.05973(0.05713~0.06233) | 0.05339(0.05143~0.05536) | -0.004(-0.007~-0.000) | 211148 | .032 |
|  | UA Fix. number | 0.4051(0.3099~0.5004) | 0.1264(0.1081~0.1446) | -6.876e-05(-6.340e-05~-3.307e-05) | 736460 | <.001 |
|  | SGE | 0.3979(0.3885~0.4073) | 0.3917(0.3854~0.398) | 7.105e-05(-6.427e-05~3.293e-05) | 824131 | .916 |
|  | GTE | 0.06429(0.05715~0.07142) | 0.03768(0.03298~0.04237) | -3.561e-05(-5.098e-05~-5.283e-05) | 746234 | <.001 |
| **Antisaccade** | Total duration (ms) | 8211.0(7730.0~8691.0) | 2667.0(2516.0~2817.0) | -2883.000(-3467.000~-2383.000) | 137389 | <.001 |
|  | TA Fix. incidence | 0.8165(0.7939~0.8391) | 0.9614(0.9515~0.9713) | 1.834e-05(7.648e-05~4.702e-05) | 937626 | <.001 |
|  | TA Fix. latency (ms) | 3495(3194.0~3796.0) | 1195(1139.0~1251.0) | -669.000(-769.000~-579.000) | 352196 | <.001 |
|  | Fix. number | 18.61(17.46~19.76) | 6.315(6.06~6.57) | -5.000(-6.000~-5.000) | 399923 | <.001 |
|  | Fix. duration average (ms) | 282.2(274.9~289.5) | 296.1(288.8~303.5) | 3.714(-3.333~10.863) | 825724 | .303 |
|  | Sac. number | 41.11(37.35~44.87) | 9.846(8.893~10.8) | -11.000(-13.000~-10.000) | 350199 | <.001 |
|  | Sac. duration average (ms) | 30.66(29.86~31.45) | 41.20(40.40~42.00) | 10.802(9.686~11.917) | 1141086 | <.001 |
|  | Sac. velocity average (°/s) | 124.3(119.3, 129.3) | 155.7(152.2, 159.2) | 39.145(34.170~44.120) | 1083742 | <.001 |
|  | Sac. peak velocity (°/s) | 183.3(175.5, 191.2) | 244.4(238.4, 250.3) | 72.925(64.065~81.865) | 1095635 | <.001 |
|  | Sac. amplitude average (°) | 8.615(8.316~8.914) | 9.889(9.614~10.16) | 1.246(0.888~1.603) | 844785 | <.001 |
|  | Pupil diameter SD (mm) | 0.1329(0.1284~0.1375) | 0.09418(0.09165~0.09671) | -0.030(-0.037~-0.024) | 131108 | <.001 |
|  | Pupil diameter MAX (mm) | 3.467(3.435~3.499) | 3.413(3.386~3.44)] | 0.066(-0.118~-0.015) | 174210 | .012 |
|  | Pupil diameter MIN (mm) | 2.944(2.916~2.972) | 3.085(3.06~3.11) | 0.130(0.089~0.170) | 228840 | <.001 |
|  | UA Fix. number | 2.811(2.516~3.106) | 0.5558(0.49~0.6216) | -1.000(-1.000~-0.000) | 530604 | <.001 |
|  | SA Fix. number | 2.924(2.676~3.172) | 0.9153(0.8686~0.962) | -1.000(-1.000~-1.000) | 484530 | <.001 |
|  | PSA Fix. incidence | 0.9609(0.9496~0.9723) | 0.9979(0.9956~1.0) | 2.291e-05(-5.768e-05~7.156e-05) | 828893 | <.001 |
|  | WSA Fix. incidence | 0.9310(0.9161~0.9459) | 0.8691(0.8518~0.8865) | -4.534e-05(-4.837e-05~-1.537e-05) | 749907 | <.001 |
|  | PSA Fix. first incidence | 0.3206(0.2932~0.3481) | 0.3891(0.364~0.4142) | 3.948e-05(6.456e-05~4.999e-05) | 854081 | <.001 |
|  | SGE | 0.6003(0.5902~0.6104) | 0.5547(0.5478~0.5616) | -0.054(-0.067~-0.039) | 639580 | <.001 |
|  | GTE | 0.2507(0.2413~0.26) | 0.1371(0.1306~0.1435) | -0.123(-0.133~-0.111) | 474067 | <.001 |
|  | Search incidence | 0.3285(0.3009~0.3561) | 0.1377(0.12~0.1555) | -1.383e-05(-3.780e-05~-1.293e-05) | 652696 | <.001 |
|  | Search number | 1.567(1.511~1.623) | 1.110(1.089~1.131) | -2.076e-05(-2.360e-05~-2.698e-05) | 26722 | <.001 |
|  | Search duration (ms) | 3257.0(3074.0~3440.0) | 2361.0(2274.0~2449.0) | -341.000(-623.000~-84.000) | 31588 | .008 |
| **Delayed saccade** | Total duration (ms) | 4110.0(4109.0~4111.0) | 4089.0(4083.0~4096.0) | -1.000(-15.000~-1.000) | 198252 | <.001 |
|  | TA-P Fix. incidence | 0.2177(0.1936~0.2419) | 0.5179(0.4922~0.5436) | 1.744e-05(4.638e-05~1.145e-05) | 1053414 | <.001 |
|  | TA-P Fix. latency (ms) | 837.0(805.0~870.0) | 578.0(564.0~592.0) | -140.000(-191.000~-93.000) | 66033 | <.001 |
|  | Fix. number | 7.583(7.39~7.776) | 6.686(6.542~6.83) | -1.000(-1.000~-1.000) | 637736 | <.001 |
|  | Fix. duration average (ms) | 341.2(328.1~354.4) | 488.3(471.2~505.3) | 114.176(101.945~126.524) | 1084779 | <.001 |
|  | Sac. number | 18.69(16.98~20.4) | 11.35(10.65~12.04) | -3.000(-4.000~3.000) | 558345 | <.001 |
|  | Sac. duration average (ms) | 25.40(24.77~26.04) | 29.03(28.48~29.57) | 3.511(2.700~4.333) | 931344 | <.001 |
|  | Sac. velocity average (°/s) | 102.5(97.42, 107.7) | 110.8(107.0, 114.6) | 9.090(5.960~12.260) | 883602 | <.001 |
|  | Sac. peak velocity (°/s) | 141.2(134.4, 148.0) | 158.2(152.6, 163.7) | 14.435(9.470~19.490) | 882860 | <.001 |
|  | Sac. amplitude average (°) | 6.402(6.121~6.682) | 6.443(6.239~6.647) | 0.241(-0.062~0.545) | 704966 | .118 |
|  | Pupil diameter SD (mm) | 0.1357(0.1316~0.1399) | 0.1245(0.1217~0.1273) | -0.007(-0.013~-0.001) | 146169 | .015 |
|  | TA-W Fix. number | 0.9427(0.8853~1.0) | 0.6543(0.6125~0.696) | -4.797e-05(-4.630e-05~-7.799e-05) | 683841 | <.001 |
|  | Intrusive Sac. incidence | 0.3065(0.2795~0.3334) | 0.1198(0.1031~0.1366) | -5.769e-05(-1.737e-05~-6.607e-05) | 659016 | <.001 |
|  | SGE | 0.3923(0.3794~0.4051) | 0.4023(0.3915~0.4131) | 3.408e-05(-4.582e-05~1.724e-05) | 786560 | .339 |
|  | GTE | 0.1958(0.1872~0.2044) | 0.1923(0.1848~0.1998) | -3.755e-05(-4.482e-05~5.802e-05) | 758870 | .552 |
