# Supplementary material for: Auxiliary Diagnosis of Children With Attention-Deficit/Hyperactivity Disorder Using Eye-Tracking and Digital Biomarkers: Case-Control Study
Source: JMIR Mhealth Uhealth. 2024 Nov 29;12:e58927. doi: 10.2196/58927 (PMC11645504; doi:10.2196/58927)
Supplement: Multimedia Appendix 4 [file mhealth_v12i1e58927_app4.docx]

**Appendix 4. Differences in eye-movement metrics between ADHD and TD groups in the antisaccade and delayed saccade tasks with different target eccentricities.**

| **Task types** | **Digital biomarkers** | **Target eccentri-city** | **ADHD Mean(95%CI)** | **TD Mean(95%CI)** | **Confidence interval for median differences Difference (95%CI for Difference)** | ***U*** | ***P*** |
| --- | --- | --- | --- | --- | --- | --- | --- |
| **Antisaccade** | PSA Fix. incidence | 7° | 0.9537(0.9322~0.9752) | 0.9979(0.9939~1.002) | 1.417e-05(-9.121e-06~4.879e-06) | 92745 | <.001 |
|  |  | 15° | 0.9754(0.9596~0.9912) | 0.9979(0.9939~1.002) | 4.937e-05(-4.951e-05~6.469e-05) | 90567 | .003 |
|  |  | 20° | 0.9538(0.9324~0.9752) | 0.9979(0.9939~1.002) | 2.068e-05(-2.054e-05~3.058e-05) | 92986 | <.001 |
|  | WSA Fix. incidence | 7° | 0.9428(0.919~0.9666) | 0.9318(0.9093~0.9544) | -3.374e-05(-3.064e-05~3.180e-05) | 87841 | .516 |
|  |  | 15° | 0.9208(0.8932~0.9483) | 0.8388(0.806~0.8717) | -9.189e-07(-2.376e-05~-2.484e-05) | 81316 | <.001 |
|  |  | 20° | 0.9293(0.9032~0.9555) | 0.8368(0.8037~0.8698) | -5.025e-05(-9.658e-06~-5.337e-05) | 80812 | <.001 |
|  | PSA Fix. first incidence | 7° | 0.3406(0.2921~0.3891) | 0.3657(0.3226~0.4088) | 1.106e-05(-2.332e-06~3.440e-05) | 91044 | .449 |
|  |  | 15° | 0.3033(0.2563~0.3502) | 0.405(0.3611~0.4488) | 5.237e-05(3.777e-05~2.262e-05) | 97578 | .002 |
|  |  | 20° | 0.3179(0.2705~0.3654) | 0.3967(0.353~0.4404) | 1.989e-05(1.386e-05~4.358e-05) | 96070 | .018 |
|  | SA Fix. number | 7° | 3.814(3.256~4.371) | 1.236(1.152~1.319) | -1.000(-1.000~-9.999e-01) | 56647 | <.001 |
|  |  | 15° | 2.971(2.57~3.371) | 0.8079(0.7341~0.8816) | -1.000(-1.000~-9.999e-01) | 46938 | <.001 |
|  |  | 20° | 1.987(1.723~2.25) | 0.7025(0.625~0.7799) | -1.000(-1.000~-9.999e-01) | 55322 | <.001 |
|  | SGE | 7° | 0.6057(0.5887~0.6227) | 0.5827(0.5725~0.5929) | -0.028(-0.048~-0.010) | 77187 | <.001 |
|  |  | 15° | 0.6032(0.585~0.6214) | 0.5383(0.5251~0.5516) | -0.079(-0.100~-0.057) | 64716 | <.001 |
|  |  | 20° | 0.5921(0.5749~0.6093) | 0.543(0.531~0.5551) | -0.054(-0.079~-0.028) | 71655 | <.001 |
|  | GTE | 7° | 0.2584(0.2412~0.2757) | 0.1323(0.1201~0.1446) | -0.133(-0.158~-0.111) | 51097 | <.001 |
|  |  | 15° | 0.2612(0.2451~0.2774) | 0.1411(0.1303~0.1519) | -0.132(-0.148~-0.111) | 50322 | <.001 |
|  |  | 20° | 0.2324(0.217~0.2478) | 0.1378(0.1274~0.1482) | -0.102(-0.121~-0.078) | 56689 | <.001 |
| **Delayed saccade** | TA-P Fix. incidence | 7° | 0.207(0.1659~0.2481) | 0.314(0.2726~0.3555) | 4.536e-05(1.233e-05~3.174e-05) | 99662 | <.001 |
|  |  | 15° | 0.2473(0.2035~0.2911) | 0.595(0.5512~0.6389) | 1.519e-06(6.862e-06~4.668e-05) | 121328 | <.001 |
|  |  | 20° | 0.1989(0.1584~0.2395) | 0.6446(0.6018~0.6874) | 1.000(5.964e-06~9.999e-01) | 130148 | <.001 |
|  | TA-P Fix. latency (ms) | 7° | 847(790.0~904.0) | 535(517.0~553.0) | -191.269(-316.000~-82.000) | 3984.5 | <.001 |
|  |  | 15° | 841(782.0~899.0) | 596(569.0~622.0) | -113.000(-189.000~ -48.000) | 9967.5 | <.001 |
|  |  | 20° | 823(769.0~876.0) | 582(559.0~606.0) | -144.000(-229.000~ -68.000) | 8205.5 | <.001 |
|  | TA-W Fix. number | 7° | 1.075(0.9737~1.177) | 0.9277(0.8545~1.001) | -2.55815e-05(-7.932e-06~8.472e-05) | 84453 | .099 |
|  |  | 15° | 1.03(0.9293~1.13) | 0.5434(0.4749~0.6119) | -7.780e-06(-9.999e-01~-4.064e-05) | 64808 | <.001 |
|  |  | 20° | 0.7231(0.6298~0.8165) | 0.4917(0.4224~0.5611) | -8.302e-05(-9.223e-05~-1.022e-05) | 77942 | <.001 |
