# Supplementary material for: Auxiliary Diagnosis of Children With Attention-Deficit/Hyperactivity Disorder Using Eye-Tracking and Digital Biomarkers: Case-Control Study
Source: JMIR Mhealth Uhealth. 2024 Nov 29;12:e58927. doi: 10.2196/58927 (PMC11645504; doi:10.2196/58927)
Supplement: Multimedia Appendix 5 [file mhealth_v12i1e58927_app5.docx]

**Appendix 5. Differences in eye-movement metrics between ADHD and TD groups for different age groups.**

| **Task types** | **Digital biomarkers** | **Age Group** | | **ADHD Mean(95%CI)** | **TD Mean(95%CI)** | **Confidence interval for median differences Difference (95%CI for Difference)** | ***U*** | ***P*** |
| --- | --- | --- | --- | --- | --- | --- | --- | --- |
| **Prosaccade** | Total duration (ms) | | Group1 | 1699.0(1378.0~2019.0) | 910.4(834.5~986.3) | -34.000(-84.000~-3.379e-05) | 26588 | .025 |
|  |  | | Group2 | 1575(1354.0~1796.0) | 1250.0(1125.0~1374.0) | -0.100(-50.000~34.000) | 26506 | .737 |
|  |  | | Group3 | 1034.0(877.3~1192.0) | 850.3(737.5~963.1) | -17.000( -50.000~5.909e-05) | 27990 | .108 |
|  | Sac. velocity average (°/s) | | Group1 | 135.5(127.2, 143.7) | 161.2(155.3, 167.0) | 32.070(23.355~40.820) | 143298 | <.001 |
|  |  |  | Group2 | 134.4(125.2, 143.5) | 159.2(152.3, 166.2) | 28.910(20.420~37.680) | 148496 | <.001 |
|  |  |  | Group3 | 131.6(123.3, 139.9) | 158.8(152.3, 165.4) | 29.200(18.595~40.150) | 61479 | <.001 |
|  | Sac. amplitude average (°) | | Group1 | 7.539(7.092~7.986) | 9.367(8.918~9.816) | 1.712(1.080~2.325) | 117804 | <.001 |
|  |  |  | Group2 | 8.428(7.943~8.914) | 8.499(8.088~8.911) | 0.190(-0.400~0.802) | 101465 | .518 |
|  |  |  | Group3 | 8.210(7.607~8.814) | 8.282(7.827~8.737) | 0.185(-0.510~ 0.880) | 40964 | .600 |
|  | Sac. Peak Velocity (°/s) | | Group1 | 201.3(189.3, 213.3) | 250.0(239.7, 260.2) | 54.950(39.845~70.180) | 143601 | <.001 |
|  |  |  | Group2 | 200.7(187.7, 213.8) | 241.6(230.7, 252.5) | 45.910(31.080~60.590) | 146352 | <.001 |
|  |  |  | Group3 | 202.2(187.8, 216.7) | 248.6(237.6, 259.5) | 52.149(33.530~70.470) | 61633 | <.001 |
| **Antisaccade** | Total duration (ms) | | Group1 | 8475.0(7728.0~9222.0) | 3616.0(3248.0~3983.0) | -2682.059(-3916~-1850) | 16074 | <.001 |
|  |  | | Group2 | 9351.0(8487.0~10220.0) | 2923.0(2688.0~3158.0) | -3600(-5200~-2366) | 12276 | <.001 |
|  |  | | Group3 | 5429.0(4685.0~6174.0) | 2155.0(1954.0~2356.0) | -1132(-1950~-700) | 17298 | <.001 |
|  | TA Fix. incidence | | Group1 | 0.7708(0.7310~0.8106) | 0.9678(0.9527~0.9829) | 5.517e-05(1.106e-05~1.861e-05) | 136512 | <.001 |
|  |  | | Group2 | 0.8086(0.7718~0.8453) | 0.9583(0.9412~0.9754) | 5.669e-05(7.124e-05~5.628e-05) | 134772 | <.001 |
|  |  | | Group3 | 0.9087(0.8729~0.9445) | 0.9571(0.9370~0.9771) | 5.533e-05(-2.47e-05~8.197e-06) | 52308 | .013 |
|  | TA Fix. latency (ms) | | Group1 | 3772.0(3311.0~4233.0) | 1330.0(1233.0~1427.0) | -831.0(-1013~-653.0) | 47651 | <.001 |
|  |  |  | Group2 | 3656.0(3141.0~4171.0) | 1188.0(1095.0~1281.0) | -692.0(-853.0~-553.0) | 47300 | <.001 |
|  |  |  | Group3 | 2841.0(2231.0~3450.0) | 1022.0(919.7~1125.0) | -462.0(-592.0~-357.0) | 23959 | <.001 |
|  | Pupil diameter SD (mm) | | Group1 | 0.1407(0.1333~0.1481) | 0.09923(0.09528~0.1032) | -0.032(-0.045~-0.020) | 13218 | <.001 |
|  |  |  | Group2 | 0.1355(0.1277~0.1433) | 0.08989(0.08593~0.09384) | -0.034(-0.047~-0.022) | 9253 | <.001 |
|  |  |  | Group3 | 0.1096(0.1019~0.1172) | 0.09395(0.08889~0.09900) | -0.014(-0.024~-0.003) | 17366 | .011 |
|  | UA Fix. number | | Group1 | 2.366(1.972~2.760) | 0.7197(0.5750~0.8644) | -1.360e-06(-1.000 -2.366e-05) | 78157 | <.001 |
|  |  | | Group2 | 3.194(2.690~3.697) | 0.4583(0.3756~0.5410) | -1.000(-1.000~-9.999e-01) | 69995 | <.001 |
|  |  | | Group3 | 2.901(2.193~3.608) | 0.4672(0.3752~0.5592) | -2.2716e-05(-1.000~-0.000) | 33941 | <.001 |
|  | SA Fix. number. | | Group1 | 3.477(3.031~3.923) | 1.053(0.9660~1.140) | -1.000(-1.000~-9.999e-01) | 64021 | <.001 |
|  |  | | Group2 | 2.890(2.486~3.293) | 0.8769(0.8038~0.9500) | -1.000(-1.000~-9.999e-01) | 67631 | <.001 |
|  |  | | Group3 | 2.036(1.673~2.399) | 0.7828(0.7053~0.8604) | 1.000(-1.000~-4.102e-05) | 33281 | <.001 |
|  | SGE | | Group1 | 0.6115(0.5955~0.6275) | 0.5744(0.5635~0.5853) | -0.0438(-0.066~-0.026) | 91465 | <.001 |
|  |  | | Group2 | 0.6012(0.5846~0.6178) | 0.5406(0.5289~0.5522) | -0.072(-0.090~-0.052) | 83979 | <.001 |
|  |  | | Group3 | 0.5798(0.5593~0.6003) | 0.5473(0.5336~0.5610) | -0.031(-0.064~-0.010) | 42544 | .002 |
|  | GTE | | Group1 | 0.2597(0.2446~0.2749) | 0.1515(0.1406~0.1623) | -0.115(-0.134~-0.096) | 67335 | <.001 |
|  |  | | Group2 | 0.2510(0.2360~0.2660) | 0.1358(0.1251~0.1465) | -0.127(-0.141~-0.107) | 67106 | <.001 |
|  |  | | Group3 | 0.2347(0.2146~0.2547) | 0.1195(0.1076~0.1315) | -0.125(-0.145~-0.095) | 28928 | <.001 |
| **Delayed saccade** | TA-P Fix. incidence | | Group1 | 0.1806(0.1441~0.2170) | 0.4508(0.4082~0.4933) | 2.930e-06(5.511e-06 4.500e-07) | 144864 | <.001 |
|  |  | | Group2 | 0.2387(0.1989~0.2785) | 0.5568(0.5143~0.5993) | 5.679e-05(6.973e-05~1.273e-05) | 154500 | <.001 |
|  |  | | Group3 | 0.2458(0.1923~0.2994) | 0.5556(0.5064~0.6047) | 6.110e-05(5.279e-06~5.523e-05) | 62238 | <.001 |
|  | TA-P Fix. latency (ms) | | Group1 | 912.0(852.0~972.0) | 584.0(561.0~607.0) | -196(-315.000~-96.000) | 6339.5 | <.001 |
|  |  |  | Group2 | 871.0(822.0~920.0) | 587.0(563.0~610.0) | -200(-275~-125) | 9941.5 | <.001 |
|  |  |  | Group3 | 677.0(620.0~735.0) | 559.0(534.0~585.0) | -30.000(-87.000~26.000) | 5939.5 | .318 |
|  | TA-W Fix. number | | Group1 | 1.056(0.9610~1.150) | 0.7652(0.6919~0.8384) | -4.060e-05(-4.523e-06~-8.410e-07) | 96053 | <.001 |
|  |  | | Group2 | 0.8919(0.7995~0.9843) | 0.6023(0.5348~0.6698) | -1.088e-05(-5.173e-05~-2.611e-05) | 99301 | <.001 |
|  |  | | Group3 | 0.8333(0.7195~0.9472) | 0.5758(0.5006~0.6509) | -2.547e-05(4.858e-05~-1.135e-05) | 40482 | <.001 |
|  | Intrusive Sac. incidence | | Group1 | 0.3611(0.3156~0.4066) | 0.1383(0.1087~0.1678) | -9.821e-06(-7.088e-05 -6.899e-05) | 88632 | <.001 |
|  |  |  | Group2 | 0.2883(0.2460~0.3306) | 0.09470(0.06964~0.1198) | -4.670e-05(-3.707e-05~-6.494e-05) | 94524 | <.001 |
|  |  |  | Group3 | 0.2417(0.1884~0.2949) | 0.1288(0.09565~0.1619) | -3.034e-05(-1.064e-05~-1.907e-05) | 42156 | <.001 |
