# Supplementary material for: Auxiliary Diagnosis of Children With Attention-Deficit/Hyperactivity Disorder Using Eye-Tracking and Digital Biomarkers: Case-Control Study
Source: JMIR Mhealth Uhealth. 2024 Nov 29;12:e58927. doi: 10.2196/58927 (PMC11645504; doi:10.2196/58927)
Supplement: Multimedia Appendix 6 [file mhealth_v12i1e58927_app6.docx]

**Appendix 6. Differences in eye-movement metrics between different age groups for ADHD and TD groups.**

| **Task** | **Features** | **ADHD/TD** | **Group1 Mean(95%CI)** | **Group2 Mean(95%CI)** | **Group3 Mean(95%CI)** | **Inter-**  **group *H*_2_** | **Inter-**  **group**  ***P*** |
| --- | --- | --- | --- | --- | --- | --- | --- |
| **Prosaccade** | Total duration (ms) | ADHD | 1699.0(1378.0~2019.0) | 1575(1354.0~1796.0) | 1034.0(877.3~1192.0) | 8.263 | .016 |
|  |  | TD | 910.4(834.5~986.3) | 1250.0(1125.0~1374.0) | 850.3(737.5~963.1) | 22.219 | <.001 |
|  | Sac. velocity average (°/s) | ADHD | 135.5(127.2, 143.7) | 134.4(125.2, 143.5) | 131.6(123.3, 139.9) | 1.061 | .588 |
|  |  | TD | 161.2(155.3, 167.0) | 159.2(152.3, 166.2) | 158.8(152.3, 165.4) | 2.616 | .270 |
|  | Sac. amplitude average (°) | ADHD | 7.539(7.092~7.986) | 8.428(7.943~8.914) | 8.210(7.607~8.814) | 5.818 | .055 |
|  |  | TD | 9.367(8.918~9.816) | 8.499(8.088~8.911) | 8.282(7.827~8.737) | 9.901 | .007 |
|  | Sac. peak velocity (°/s) | ADHD | 201.3(189.3, 213.3) | 200.7(187.7, 213.8) | 202.2(187.8, 216.7) | 1.0528 | .591 |
|  |  | TD | 250.0(239.7, 260.2) | 241.6(230.7, 252.5) | 248.6(237.6, 259.5) | 4.5257 | .104 |
| **Antisaccade** | Total duration (ms) | ADHD | 8475.0(7728.0~9222.0) | 9351.0(8487.0~10220.0) | 5429.0(4685.0~6174.0) | 30.199 | <.001 |
|  |  | TD | 3616.0(3248.0~3983.0) | 2923.0(2688.0~3158.0) | 2155.0(1954.0~2356.0) | 35.212 | <.001 |
|  | TA Fix. incidence | ADHD | 0.7708(0.7310~0.8106) | 0.8086(0.7718~0.8453) | 0.9087(0.8729~0.9445) | 20.488 | <.001 |
|  |  | TD | 0.9678(0.9527~0.9829) | 0.9583(0.9412~0.9754) | 0.9571(0.9370~0.9771) | 0.917 | .632 |
|  | TA Fix. latency (ms) | ADHD | 3772.0(3311.0~4233.0) | 3656.0(3141.0~4171.0) | 2841.0(2231.0~3450.0) | 18.394 | <.001 |
|  |  | TD | 1330.0(1233.0~1427.0) | 1188.0(1095.0~1281.0) | 1022.0(919.7~1125.0) | 44.249 | <.001 |
|  | Pupil diameter SD (mm) | ADHD | 0.1407(0.1333~0.1481) | 0.1355(0.1277~0.1433) | 0.1096(0.1019~0.1172) | 14.354 | <.001 |
|  |  | TD | 0.09923(0.09528~0.1032) | 0.08989(0.08593~0.09384) | 0.09395(0.08889~0.09900) | 4.623 | .009 |
|  | UA Fix. number | ADHD | 2.366(1.972~2.760) | 3.194(2.690~3.697) | 2.901(2.193~3.608) | 3.367 | .186 |
|  |  | TD | 0.7197(0.5750~0.8644) | 0.4583(0.3756~0.5410) | 0.4672(0.3752~0.5592) | 5.363 | .068 |
|  | SA Fix. number | ADHD | 3.477(3.031~3.923) | 2.890(2.486~3.293) | 2.036(1.673~2.399) | 27.396 | <.001 |
|  |  | TD | 1.053(0.966~1.140) | 0.8769(0.8038~0.9500) | 0.7828(0.7053~0.8604) | 22.371 | <.001 |
|  | SGE | ADHD | 0.6115(0.5955~0.6275) | 0.6012(0.5846~0.6178) | 0.5798(0.5593~0.6003) | 7.35 | .025 |
|  |  | TD | 0.5744(0.5635~0.5853) | 0.5406(0.5289~0.5522) | 0.5473(0.5336~0.5610) | 16.482 | <.001 |
|  | GTE | ADHD | 0.2597(0.2446~0.2749) | 0.2510(0.2360~0.2660) | 0.2347(0.2146~0.2547) | 4.311 | .116 |
|  |  | TD | 0.1515(0.1406~0.1623) | 0.1358(0.1251~0.1465) | 0.1195(0.1076~0.1315) | 13.773 | .001 |
| **Delayed saccade** | TA-P Fix. incidence | ADHD | 0.1806(0.1441~0.2170) | 0.2387(0.1989~0.2785) | 0.2458(0.1923~0.2994) | 5.763 | .056 |
|  |  | TD | 0.4508(0.4082~0.4933) | 0.5568(0.5143~0.5993) | 0.5556(0.5064~0.6047) | 14.975 | <.001 |
|  | TA-P Fix. latency (ms) | ADHD | 912.0(852.0~972.0) | 871.0(822.0~920.0) | 677.0(620.0~735.0) | 8.652 | .013 |
|  |  | TD | 584.0(561.0~607.0) | 587.0(563.0~610.0) | 559.0(534.0~585.0) | 2.101 | .350 |
|  | TA-W Fix. number | ADHD | 1.056(0.9610~1.150) | 0.8919(0.7995~0.9843) | 0.8333(0.7195~0.9472) | 10.547 | .005 |
|  |  | TD | 0.7652(0.6919~0.8384) | 0.6023(0.5348~0.6698) | 0.5758(0.5006~0.6509) | 15.785 | <.001 |
|  | Intrusive Sac. incidence | ADHD | 0.3611(0.3156~0.4066) | 0.2883(0.2460~0.3306) | 0.2417(0.1884~0.2949) | 11.491 | .003 |
|  |  | TD | 0.1383(0.1087~0.1678) | 0.09470(0.06964~0.1198) | 0.1288(0.09565~0.1619) | 5.16 | .076 |
