# Supplementary material for: Auxiliary Diagnosis of Children With Attention-Deficit/Hyperactivity Disorder Using Eye-Tracking and Digital Biomarkers: Case-Control Study
Source: JMIR Mhealth Uhealth. 2024 Nov 29;12:e58927. doi: 10.2196/58927 (PMC11645504; doi:10.2196/58927)
Supplement: Multimedia Appendix 7 [file mhealth_v12i1e58927_app7.docx]

**Appendix 7. Validation of the impact of down-sampling on model training.**

for future compatibility of this method using portable eye trackers, the predictive effectiveness of the model at low sampling rates was validated. 120 Hz and 300 Hz were selected as the common sampling rates for portable eye-tracking devices currently on the market.

we recruited a group of clinically diagnosed ADHD and TD children from a developmental-behavioral pediatric outpatient clinic. We acquired data using the same task at a lower sampling frequency, and the data were used to validate our model.

We collected data from two additional groups of participants: 23 participants (19 males and 4 females) comprised the 120 Hz group and 26 participants (22 males and 4 females) comprised the 300 Hz group. There were no differences in age, clinical diagnosis, IQ, or SNAP-IV scores between the two groups. The following are their basic information.

**Appendix Table 7.1. The basic information of the participants that performed data acquisition using a low sampling rate.**

|  | Variable | 120Hz  (N=23) | 300Hz  (N=26) | *t* or *chi-*  *square* value | *P*-value |
| --- | --- | --- | --- | --- | --- |
| **Sex** | | | | | |
|  | Male, n (%) | 19(82.6) | 22(84.6) | 0.04 | .850 |
|  | Female, n (%) | 4(17.4) | 4(15.4) |  |  |
| **Age** | | | | | |
|  | Age | 8.31±1.28 | 8.54±1.14 | 0.67 | .509 |
| **Clinical Diagnosis** | |  |  |  |  |
|  | TD, n (%) | 13(56.5) | 11(42.3) | 0.75 | .386 |
|  | ADHD, n (%) | 10(43.5) | 14(53.8) |  |  |
| **IQ (Intelligence Quotient)** | | | | | |
|  | Verbal IQ (mean±SD) | 90.26±10.28 | 92.81±8.13 | 0.97 | .338 |
|  | Performance IQ (mean±SD) | 95.83±9.46 | 94.69±11.10 | 0.38 | .703 |
|  | Full Scale IQ (mean±SD) | 91.83±8.45 | 92.81±8.15 | 0.41 | .682 |
| **SNAP-IV** | | | | | |
|  | Inattentive | 13.22±6.30 | 13.65±5.83 | 0.25 | .805 |
|  | Hyperactivity/Impulsive | 10.43±6.30 | 11.04±5.04 | 0.38 | .709 |
|  | Oppositional Defiant | 7.43±6.18 | 10.04±6.15 | 1.48 | .146 |

According to the results of the validation study, although the use of external data resulted in slightly lower evaluation metrics, the overall performance was consistent with the original model. The results demonstrated that our model adapted well to low-sampling rate data, which further confirms its high generalizability and applicability to portable devices.

**Appendix Table 7.2. The evaluation metrics of the classification model trained with low sampling rate.**

|  | AUC | Accuracy | Sensitivity | Specificity | Precision | F1 score |
| --- | --- | --- | --- | --- | --- | --- |
| The origin model | **0.965** | **0.908** | **0.877** | **0.932** | **0.913** | **0.892** |
| 300Hz | **0.861** | **0.870** | **0.900** | **0.846** | **0.818** | **0.857** |
| 120Hz | **0.881** | **0.885** | **0.857** | **0.917** | **0.923** | **0.889** |
