# Supplementary material for: Auxiliary Diagnosis of Children With Attention-Deficit/Hyperactivity Disorder Using Eye-Tracking and Digital Biomarkers: Case-Control Study
Source: JMIR Mhealth Uhealth. 2024 Nov 29;12:e58927. doi: 10.2196/58927 (PMC11645504; doi:10.2196/58927)
Supplement: Multimedia Appendix 8 [file mhealth_v12i1e58927_app8.docx]

**Appendix 8. Validation of the effect of sex differences on model training.**

Due to the variability in ADHD prevalence and tendency to visit the clinic across sex, there was a large difference in the sex ratio of ADHD participants included in this study. To verify the role of sex differences in model training, two models were trained using data from boys and girls separately and validating using data of the opposite sex. The evaluation metrics of the two models were compared with those of the original model to verify whether the variability between sexes affected model construction.

Despite the smaller sample size, we found that the two models trained using single-sex data performed reasonably well; moreover, the model trained using male data achieved higher performance for predicting female data than the original model. This result suggests that sex does not significantly affect modeling, and the current model trained with predominantly male data can also be applied to predict female subjects.

**Appendix Table 8.1. The evaluation metrics of the classification model trained by single-sex samples.**

| Model | AUC | Accuracy | Sensitivity | Specificity | Precision | F1 score |
| --- | --- | --- | --- | --- | --- | --- |
| The origin model | **0.965** | **0.908** | **0.877** | **0.932** | **0.913** | **0.892** |
| Training on male samples and validating on female samples | **0.970** | **0.930** | **0.800** | **0.951** | **0.727** | **0.762** |
| Training on female samples and validating on male samples | **0.966** | **0.807** | **0.679** | **0.984** | **0.983** | **0.803** |
